# Projecting prevalence of frailty and dementia and the economic cost of care in Japan from 2016 to 2043: a microsimulation modelling study

**DOI:** 10.1016/S2468-2667(22)00044-5

**Published:** 2022-05

**Authors:** Megumi Kasajima, Karen Eggleston, Shoki Kusaka, Hiroki Matsui, Tomoki Tanaka, Bo-Kyung Son, Katsuya Iijima, Kazuo Goda, Masaru Kitsuregawa, Jay Bhattacharya, Hideki Hashimoto

**Affiliations:** School of Public Health, University of Tokyo, Tokyo, Japan; Walter H Shorenstein Asia-Pacific Research Center, Freeman Spogli Institute for International Studies, Stanford University, CA, USA; Graduate School of Economics, University of Tokyo, Tokyo, Japan; School of Public Health, University of Tokyo, Tokyo, Japan; Institute of Gerontology, University of Tokyo, Tokyo, Japan; Institute of Gerontology, University of Tokyo, Tokyo, Japan; Institute for Future Initiatives, University of Tokyo, Tokyo, Japan; Institute of Gerontology, University of Tokyo, Tokyo, Japan; Institute for Future Initiatives, University of Tokyo, Tokyo, Japan; Institute of Industrial Science, University of Tokyo, Tokyo, Japan; Institute of Industrial Science, University of Tokyo, Tokyo, Japan; Center for Primary Care and Outcomes Research, Stanford School of Medicine, CA, USA; School of Public Health, University of Tokyo, Tokyo, Japan

## Abstract

**Background:**

Dementia and frailty often accompany one another in older age, requiring complex care and resources. Available projections provide little information on their joint impact on future health-care need from different segments of society and the associated costs. Using a newly developed microsimulation model, we forecast this situation in Japan as its population ages and decreases in size.

**Methods:**

In this microsimulation modelling study, we built a model that simulates an individual’s status transition across 11 chronic diseases (including diabetes, coronary heart disease, and stroke) as well as depression, functional status, and self-reported health, by age, sex, and educational strata (less than high school, high school, and college and higher), on the basis of nationally representative health surveys and existing cohort studies. Using the simulation results, we projected the prevalence of dementia and frailty, life expectancy with these conditions, and the economic cost for formal and informal care over the period 2016–43 in the population of Japan aged 60 years and older.

**Findings:**

Between 2016 and 2043, life expectancy at age 65 years will increase from 23·7 years to 24·9 years in women and from 18·7 years to 19·9 years in men. Years spent with dementia will decrease from 4·7 to 3·9 years in women and 2·2 to 1·4 years in men. By contrast, years spent with frailty will increase from 3·7 to 4·0 years for women and 1·9 to 2·1 for men, and across all educational groups. By 2043, approximately 29% of women aged 75 years and older with a less than high school education are estimated to have both dementia and frailty, and so will require complex care. The expected need for health care and formal long-term care is anticipated to reach costs of US$125 billion for dementia and $97 billion for frailty per annum in 2043 for the country.

**Interpretation:**

Japan’s Government and policy makers should consider the potential social challenges in caring for a sizable population of older people with frailty and dementia, and a widening disparity in the burden of those conditions by sex and by educational status. The future burden of dementia and frailty should be countered not only by curative and preventive technology innovation, but also by social policies to mitigate the health gap.

**Funding:**

Japan Society for the Promotion of Science, Hitachi – the University of Tokyo Laboratory for a sustainable society, and the National Institute of Ageing.

## Introduction

Population ageing brings with it the challenges of age-related conditions, such as dementia and physical frailty, for which spending on health care and caregiving is expected to increase. In 2016, approximately 44 million people worldwide had dementia, almost double the number in 1990.^1^ The prevalence of frailty is rapidly increasing globally along with population ageing and will increase health-care demand.^2^

Among member countries of the Organisation for Economic Co-operation and Development, Japan stands on the front-line of population ageing. As of 2020, the Japanese population aged 65 years and older was more than 36·0 million (approximately 29·2% of the population).^3^ An estimated 3·5 million people had dementia (approximately 8% of the global burden^3^) and an estimated 3 million people had frailty in 2012.^4^

Japan has had universal public insurance coverage for health care that allows affordable access to outpatient, inpatient, and pharmaceutical care since 1961.^5^ In 2000, the Japanese Government launched a public, mandatory long-term care insurance (LTCI) scheme to support formal provision of personal and social care by trained support workers (hereafter referred to as formal care). Long-term care might be provided in care homes and chronic-care hospitals, in community centres, or at home.^6^ After the scheme’s launch, the number of total long-term care beneficiaries and associated expenditure increased substantially, despite cost control policies, from US$33 billion in 2000 to $94 billion in 2018.^7^

What will future demand for health care and long-term care be in Japan? The burden of ageing-related conditions is expected to increase in Japan in the near future, and also in other countries in Asia and in several other regions of the world where population ageing outpaces the rate seen in Japan.^8^

Use of microsimulation models allows future projection of population health by accounting for intertwined associations between health, demographics, and risk factors on an individual basis.^9,10^ However, existing microsimulation models have not articulated the joint evolution of frailty and dementia, which are two major challenges to the health-care system in an ageing population. Both are strongly associated with each other, alongside other comorbid conditions typically observed among older people.^11^ We aimed to fill this knowledge gap and estimate the economic cost of health care and long-term care services related to dementia and frailty, and disparities in these conditions, which can help inform health-care and social policy measures to better meet the challenge of population ageing.

## Methods

### Study design and construction of a multi-state transition model for multi-comorbidity

In this microsimulation modelling study, we used a recently developed microsimulation model^12^ with field-based measurement of cognitive function and frailty status to determine the association between frailty and dementia in older people (aged ≥60 years) in Japan.

Individual-based microsimulation models have been used to forecast the multi-comorbidity status of older people in England (aged ≥65 years) and the USA (aged ≥50 years), using panel data from nationwide samples of older people (appendix pp 5–7).^9,10^ We searched PubMed and Google Scholar between Jan 1 and Jan 15, 2022 for publications since database inception in English and in Japanese that forecast future disabilities related to cognitive impairment and physical frailty and their economic impact. Given the paucity of suitable panel data for the older old (aged ≥75 years) age strata in Japan,^13^ we used repeated nationwide cross-sectional surveys and death records to construct a simulated cohort: a multistate transition probabilities model of the population aged 60 years and older.^12^ The model originally used age, sex, the incidence of 11 chronic diseases (diabetes, coronary heart disease, stroke, hypertension, hyperlipidaemia, cancer of all types, respiratory diseases [including chronic obstructive pulmonary disease], joint conditions, eye problems, chronic kidney disease, and other diagnoses), two indicators of functional status (activities of daily living and instrumental activities of daily living), depression, and self-reported health. We further incorporated an education-strata-specific state transition based on education-stratified cause-specific fatalities, by using a census-death record data linkage; this is the model we used in these analyses.^14^

The current revised model starts with a baseline population aged 60 years and older and their health conditions as of June, 2016, evolving over a half-year cycle in a first-order Markov process. We used condition-specific incidence rates and case fatalities estimated for the most recent year as of 2015 for future projections. Younger cohorts (those entering the age 60–62 years strata at the time of each estimation between 2016 and 2043) were stochastically prepared on the basis of the education-stratified population (ie, less than high school education, high school education, and college education and higher) as of the 2010 census, processed with exit from the cohort due to death at the estimated age-sex-education-specific death rate until they matured to age 60 years to enter the model. Details of the model and data sources are in the appendix (pp 8–12).

### Identification of dementia and frailty status

The identification of dementia status by self-reports of physician diagnoses could lead to underestimation. Hence, previous microsimulation studies have relied on cognitive function measurements to estimate the prevalence of dementia.^15^ Similarly, we derived cognitive function measurements from the Japanese Study of Aging and Retirement (JSTAR), a sister survey to the English Longitudinal Study of Ageing and Health and Retirement Study.^16^

We measured amnestic cognitive impairment conditions on the basis of failed performance on standard cognitive tests given to JSTAR participants (namely, immediate and delayed word recall and serial-sevens examinations) and the reporting of difficulty performing at least one of seven instrumental activities of daily living (ie, using transportation, grocery shopping, preparing hot meals, paying bills, making deposits and withdrawals at the bank or using an automated teller machine (known as an ATM), using telephones, and taking medication). These items are commonly used in existing algorithms to assess dementia status using social survey data, such as the Health and Retirement Study.^10,15^

Using a multivariate probit model (appendix p 14), we estimated the likelihood of failed test performance regressed on age, sex, educational attainment, and multi-comorbidity conditions to predict dementia status for each individual in the population. We set the probability thresholds to match the prevalence of dementia and mild cognitive impairment previously reported in Japanese epidemiological surveys.^17^

There is no consensus on the standardised tools to assess frailty, which complicates status identification.^2^ We relied on data from the Kashiwa study,^18^ a population-based community survey that measured the frailty status of 1952 people aged 65 years and older in Kashiwa City, a suburb of Tokyo. This study used a modification of the Cardiovascular Health Study criteria—the most widely used method for frailty assessment.^19,20^ We constructed a logistic regression model of frailty as a function of age, sex, educational attainment, comorbid conditions (diabetes, heart disease, stroke, hypertension, hyperlipidaemia, cancer, and chronic renal failure), depression, and impaired mobility in daily living, similar to previous studies on frailty risk assessment.^21^ We extrapolated the probability of frailty in the simulated individuals with a threshold to match the real-world prevalence reported in a previous Japanese epidemiological survey.^4^ The methodological details, regression results, and estimated age-specific and sex-specific prevalence of dementia and frailty are in the appendix (pp 13–16, 26–27).

### Estimation of economic cost for health care and long-term care

On the basis of projections of the future prevalence of multi-morbidity, we estimated the economic cost of health care and formal long-term care. We defined formal care as being provided by paid professionals, and informal care as being provided by mainly unpaid non-professionals (eg, family members). We relied on electronic claim data from the National Health Insurance and LTCI databases, which contain monthly information on types of services used and volume of use.^5,21^

Japanese public health-care insurance universally covers inpatient, outpatient, and pharmaceutical services for acute and chronic conditions.^5^ Public LTCI covers nearly 90% of formal long-term care in Japan, including for home-based and community-based care and care in care homes and chronic-care hospital beds.^6^ Taking advantage of a unique reimbursement system by which fee-for-service payments are strictly based on an item-by-item price list set by the government, we applied the reference price list to administrative data to generate an estimate of the cost of health care and formal long-term care, regardless of the actual use of services.

Using nationwide administrative data on health care, we regressed monthly use by service type (inpatient *vs* outpatient and prescription) on age, sex, the 11 prespecified comorbidities, and the number of comorbid conditions. Then, we extrapolated the expected annual use for each individual in the simulated cohort population. Japan’s LTCI defines seven eligibility criteria levels according to the severity of care needs. We categorised the lower three levels as mild dependency (eg, needing to be accompanied on outings), and the higher four levels as high dependency (requiring support in activities of daily life—eg, bathing, toileting, or eating meals). We estimated mean monthly use by age-specific and sex-specific dependency strata and by service type (home-based and community-based care *vs* care in care homes) using nationwide LTCI administrative data, then extrapolated these estimates to annual use for each individual in the simulated cohort population (appendix pp 19–25).

Forecasting the economic costs of informal care provision (ie, by non-paid non-professional caregivers) is very complex because the intensity of care provision and its hourly unit price differ according to recipients’ need levels and the caregiver’s age, gender, occupational status, and relationship to the recipient (eg, spouse, child, other family member, or a friend).^22^ By 2040, a predicted 40% of households in Japan will have a single occupant, and 44% of households will be headed by someone aged 65 years or older, and most often an older couple.^23^ Given the uncertainty around who will provide informal care in the future, we provide estimates by assuming that the resource use pattern between formal and informal care will remain constant over time, with a simple sensitivity analysis. Existing literature on informal long-term care indicates that, in addition to the formal services covered by LTCI, family caregivers devote an average of 25 h per week to informal care for highly dependent people, and approximately 10 h per week for people with mild dependency.^24,25^ We multiplied these numbers by the hourly wage rate of $11 (the average for formal caregivers in 2013) and by the size of the population projected to require home-based care.

### Validation of estimated prevalence and cost of care

We did validation checks against external data for estimated prevalence and use costs. We also validated our population projections using official government forecasts.^26^ We checked health transition probabilities via backward validation, by comparing projected prevalence as of 2016 using a 2010 baseline, with actual prevalence numbers observed in the 2016 national health survey. Projections of educational attainment were validated by comparing with Barro-Lee data.^27^ We used data reported by Toshiharu Ninomiya^28^ and Takashi Asada^17^ for the age-specific and sex-specific prevalence of dementia, and of Murayama et al^4^ for those of frailty. We compared an estimated life expectancy at age 65 years as of 2016 with life expectancy using an abridged life table published by the Japanese Ministry of Health, Labour and Welfare.^29^ For formal care use, we validated the estimated results for 2016 with annual use for the same year as publicly announced by the relevant government agency (appendix pp 21, 25).

### Model outputs

The nationally representative survey we analysed—Comprehensive Survey of People’s Living Conditions—is undertaken every 3 years (appendix p 8); therefore, we adopted a transition interval of 3 years for our model. We present the prevalence of dementia and frailty for 2016, 2025, 2034, and 2043 (9 year intervals). We present estimated life expectancy free of dementia or frailty at age 65 years using the Sullivan method in the corresponding years.^30^ We also prepared the estimation by educational attainment strata and by sex, to assess socioeconomic disparity in the effect of dementia and frailty. We stochastically prepared the baseline population at age 60 years, and obtained the Monte Carlo error and 5th to 95th percentile ranges as uncertainty intervals by implementing 50 iterations of bootstrap simulation.^9^

We used Python (version 3.7.7) for parameterisation of multi-state transition probabilities and Stata SE (version 14.0) for regression analyses and simulations.

### Role of the funding source

The funders of the study had no role in study design, data collection, data analysis, data interpretation, or writing of the report.

## Results

**Figure 1:**
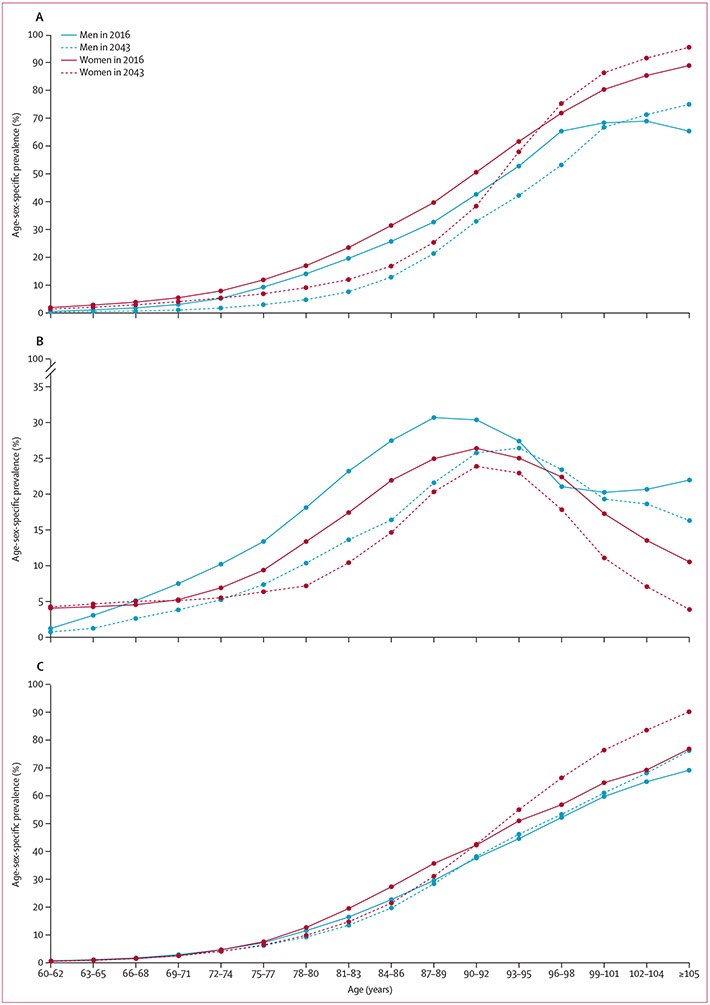
Age-sex-specific prevalence of dementia (A), mild cognitive impairment (B), and frailty (C), estimated as of 2016 and 2043, in men and women aged 60 years and older

The estimated age-specific prevalence of dementia is set to decrease between 2016 and 2043 for women younger than 95 years and for men younger than 100 years (figure 1A). For 2043 versus 2016, the prevalence peak of mild cognitive impairment is shifted into older age groups (figure 1B), indicating that in 2043 older people will have more years with cognitively normal functions.

By contrast, the prevalence of frailty in this population is estimated to increase between 2016 and 2043, specifically among those aged 90 years and older of both sexes, reflecting improved longevity and the age-dependency of the incidence of frailty (figure 1C).

We estimated the baseline prevalence of dementia among people aged 60 years and older to be approximately 5·10 million in 2016 (3·53 million women and 1·57 million men), and this prevalence is projected to be 5·03 million in 2025 (3·33 million women and 1·70 million men; table 1). In 2034, when the population of people aged 65 years and older is projected to peak, the number who have dementia is projected to remain approximately the same, at 4·90 million, followed by a gradual decrease to 4·65 million in 2043.

The projected prevalence of dementia among people aged 60–74 years shows a decreasing trend for both sexes until 2034, at which point it increases slightly. However, the projected prevalence among people aged 75 years and older differs by sex, with the prevalence among men peaking in 2025 and decreasing thereafter; whereas, a consistent increase in prevalence is seen among women, most likely because of longer life expectancy than men and an increased prevalence among people in their late 90s and older (table 1, figure 1).

The baseline prevalence of frailty in 2016 was 4·13 million (2·74 million women and 1·40 million men), and this number is expected to reach 5·24 million in 2043 (3·51 million women and 1·72 million men; table 1). The prevalence of frailty among those aged 75 years and older is projected to increase by approximately 1·3 times between 2016 and 2043 across both sexes. Notably, 1·48 million women aged 75 years and older are projected to have both dementia and frailty in 2043.

We present education-stratified prevalence data in the appendix (pp 29–31). The education-related gap in the prevalence of dementia and frailty is most notable among those aged 75 years and older, and will increase from 2016 to 2043. Approximately 29% of women aged 75 years and older with less than high school educational attainment (368 456 of 1 285 013 women) are projected to have both dementia and frailty in 2043, whereas among women in the same age group with college-level education or higher the projected prevalence is approximately 7% (293 985 of 4 553 651).

**Figure 2:**
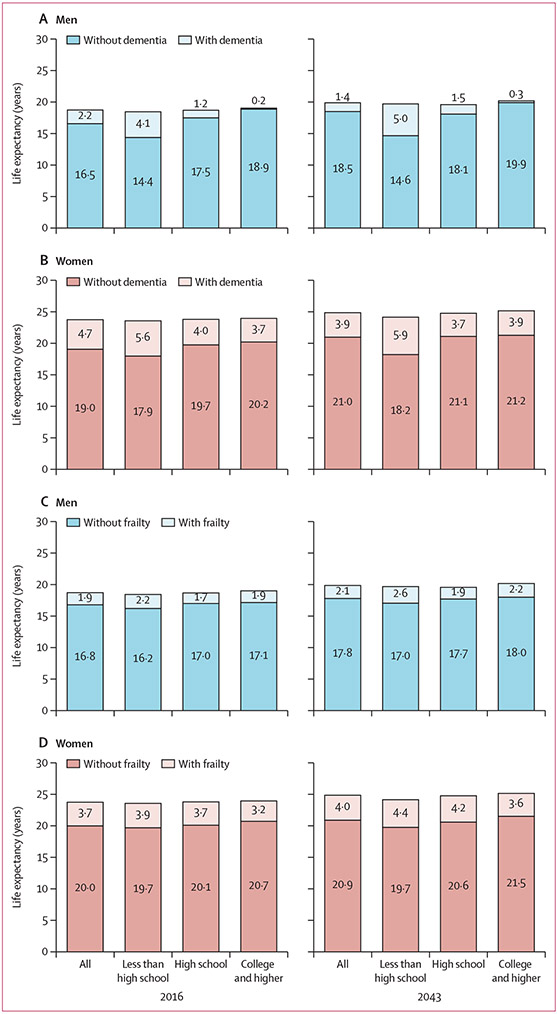
Projected life expectancy at age 65 years with and without dementia (A, B) or frailty (C, D) in 2016 and 2043, by sex and educational attainment

Our simulation indicates that life expectancy will continue to increase and the compression of years affected by dementia (figure 2A, B). Overall, between 2016 and 2043, life expectancy at age 65 years will increase from 23·7 years to 24·9 years in women and from 18·7 years to 19·9 years in men. Life expectancy with dementia at age 65 years was 4·7 years for women and 2·2 years for men in 2016, and decreased to 3·9 years in women and 1·4 years in men in 2043. Dementia-free life expectancy increases with higher educational attainment and this discrepancy is most notable in men.

We observed extension of life expectancy with frailty in both sexes between 2016 and 2043, more prominently among women than among men, with years spent with frailty increasing from 3·7 to 4·0 years for women and 1·9 to 2·1 for men (figure 2C, D).

**Figure 3:**
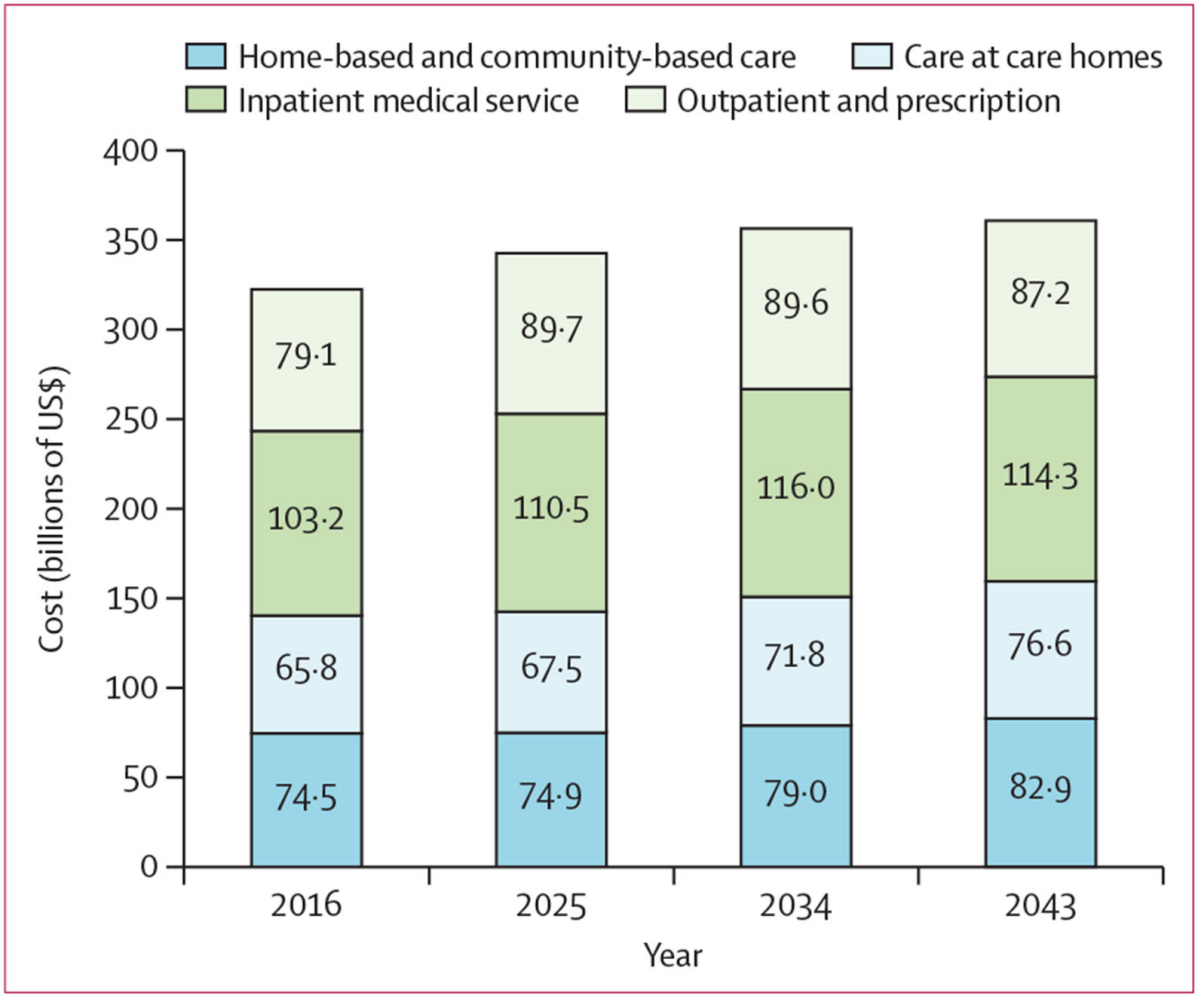
Projected costs of health care and formal long-term care for the population aged 60 years and older Health care includes outpatient and prescriptions and inpatient medical services. Formal long-term care includes home-based and community-based care and care in care homes. Annual cost estimation in billions of US$ at 2016 value.

The estimated annual cost of health care and formal long-term care for people aged 60 years and older is projected to reach $361 billion in 2043, a 12% increase from the $323 billion in 2016 (figure 3). The annual cost of health care is projected to peak in 2034, when the size of the older population also peaks, whereas the annual cost of long-term care (ie, home-based and community-based care and care in care homes) is expected to continue increasing. Health care and formal long-term care costs for people aged 60 years and older with dementia amounted to $123 billion in 2016, and is projected to increase to approximately $125 billion in 2043. Health care and long-term care costs for those with frailty amounted to $77 billion in 2016, and is projected to increase to $97 billion in 2043.

In 2016, the annual cost per capita for health care and formal long-term care among women with an educational level of less than high school was almost twice that of their counterparts with a college education or higher, and the gap widened further by 2043 (table 2). Whereas, for men the difference in annual costs between those with less than high school education and college education or higher is not as pronounced. This gap is largely attributable to the difference in estimated long-term care costs. Among those with lower educational attainment, women require more spending on formal long-term care and health care than do men, and this sex difference is projected to increase between 2016 and 2043 (table 2). Women with a lower level of education with dementia or frailty, or both, consistently bear the highest cost per capita.

Finally, we estimate the additional cost for informal care provision to be $93·1 billion in 2016, 59% of which was related to dementia care and 33% to frailty care. The cost is expected to increase to $103·3 billion in 2043, 52% of which is related to dementia care and 36% to frailty care. Because household size and the related capacity for informal care are projected to decrease by approximately 20% between 2016 and 2043, we did a sensitivity analysis assuming a substitutional increase of 13% in community-based home care, on the basis of the existing literature (appendix p 22).^31^ The results of this analysis suggest that the total formal and informal care costs will remain at approximately $460 billion per annum in all those aged 60 years and older after substitution (appendix p 22).

## Discussion

To our knowledge, this is the first microsimulation-based forecast of the joint distribution of dementia and frailty, which are major health-care challenges for the ageing population.

Despite rapid population ageing in Japan across the projected period (2016–43), the time spent with dementia is expected to decrease among those aged 60–74 years, especially among men, primarily attributable to projected improved educational attainment and reduced cardiovascular risks among that subpopulation. Previous forecasts of the prevalence of dementia, which relied on static assumptions without considering the anticipated improvement in educational attainment and cardiovascular risks among the future older population, estimated that the population with dementia would reach 9·0 million, or 25% of the population aged 65 years and older in Japan by 2040,^28,32^ a figure that exceeds our estimates. Recent estimation with consideration of risk factor improvement over time by the GBD 2019 Dementia Forecasting Collaborators projected the prevalence of dementia in Japan to be 5·2 million in 2050, which seems closer to our estimate.^33^

The impact of frailty is likely to increase for both men and women, with larger increases in the prevalence of frailty predicted for women than for men. Compared with dementia, the incidence and progression of which is strongly associated with demographic factors, physical frailty might reflect near-universal susceptibility to age-related physiological decline that could result in smaller education-related disparities and a larger burden among women than among men, given their longer life expectancy.

Existing dynamic microsimulation models for England, the USA, and other countries consistently indicate that the prevalence of dementia in the overall population will increase, despite decreasing age-specific incidence and prevalence, because of improved longevity.^9,10,33^ In Japan, decreased prevalence among those younger than 95 years, and increased prevalence thereafter, seem to counterbalance the increased number of older people with dementia caused by extended life expectancy. However, we estimate that care costs will remain high, with an increase in comorbid dementia and frailty requiring increasing costs per capita. With an expected decrease in the size of the working-age population,^23^ the social burden of care on the working-age population will become more onerous.

Our dynamic microsimulation model is similar to the PACSim project, which jointly models dementia with multiple comorbidities and socioeconomic conditions of the older population.^8^ PACSim also relies on several nationally representative surveys with a specific functional measurement obtained from the English Longitudinal Study of Ageing. Our model primarily uses crosssectional, nationally representative surveys, augmented by existing population-based survey data for measurement of cognitive functions and physical frailty.

Despite demographic and institutional differences between England and Japan, we found important similarities between the two countries. In particular, we found that the effect of dementia on requirements for caregiving will differentially affect subpopulations of older people, depending on their sex and socioeconomic conditions. An education-related gap in the prevalence of dementia has also been reported in the USA.^15^ Our forecast indicates that comorbid dementia and frailty will be more prevalent among women aged 75 years and older than other subpopulations and among those with a lower level of education than with a higher level of education, who will require greater resources for their complex care needs.

Our study has some limitations. First, the method we used for classification of dementia status has not been tested directly with the concurrent clinical measurement of cognitive function. Although our estimation successfully replicated the age-specific prevalence distribution of dementia among the Japanese population, it might misclassify status at the individual level. Moreover, the cross-country comparison and cross-validation of different classification algorithms require caution and further research.^34^ We also had to rely on urban data for field-based measurement of frailty that might not necessarily reflect rural situations. Because approximately 95% of the Japanese population lives in cities, the use of urban data might still provide a plausible projection of frailty. Second, the model does not include information on health-related behaviours, such as smoking, exercise, and dietary habits, which are known correlates of health in old age.^35-37^ In the state-transition model we adopted, we had to assume non-reversibility of chronic conditions for model simplicity; however, health behaviours will change over the life course. With limited availability of information on health behaviours in the data source, our simulation could not incorporate behavioural information, which might lead to an overestimation of the future prevalence of dementia, given, for example, the decreasing trend in smoking.^33^ Third, our model assumes constant transition probabilities between multi-comorbid statuses after 2015. Because some comorbid statuses are competing risks for others, change in one comorbid incidence will affect others in a complex way, which might lead to overestimation or underestimation. Our model also assumes constant effectiveness of curative and preventive health-care technology in the near future; an assumption which is likely to lead to the overestimation of future disease prevalence. Finally, our current study assumes constant patterns of use of formal and informal care and does not consider how health and demographic trends might affect the demand for informal care and the indirect costs of lost productivity. Therefore, our results are likely to underestimate the future economic impact of frailty and dementia.

Our projection of reduced prevalence of dementia in the next 20 years in a subset of the population is good news for a rapidly ageing but highly educated population, although this trend is unevenly distributed across the sexes and by socioeconomic status. Tertiary education and economic participation opportunities are less available to women than to men in Japan.^38^ Consequently, even with high educational attainment, women in Japan probably experience greater stress and poorer health than do men.^39^ Further research and policy discussion on whether closing the sex-related gap in education and social participation leads to a diminishing future burden of dementia and frailty in society might be warranted.

Japan’s working-age population is expected to decrease in size for the foreseeable future. An increasing fraction of the population will have decreasing functional status. Meanwhile, many promising interventions could potentially delay the onset of frailty, such as regular exercise and psychosocial support.^36^ However, in addition to individual-level interventions, Japan must invest in developing social and physical environments that are inclusive for people with such disabilities. Although Japanese people might have fewer years lived with dementia in the near future, a disproportionate concentration of frailty and dementia among susceptible subpopulations requires attention to improve health equity beyond simply regarding population ageing as a social burden. Both sex and educational disparities in health among the older population deserve concentrated attention as part of the public health policy agenda for population ageing. The future burden of dementia and frailty should be countered by curative and preventive technology innovation, and further mitigated by social policies to close the health gap.

## Data sharing

Government microdata are available by due application according to the Statistics Act article 33. JSTAR data are available on request with approval for use from the Research Institute of Economy, Trade, and Industry, Japan. Availability of Kashiwa study data is restricted due to confidentiality arrangements with the Kashiwa Municipality authority. Model codes and summary data from the study are available from the corresponding author upon request.

## Supplementary Material

1

## Figures and Tables

**Table 1:** Projected prevalence of dementia, mild cognitive impairment, and frailty in 2016, 2025, 2034, and 2043, by sex

	2016		2025		2034		2043	
	Number of cases	Prevalence proportion	Number of cases	Prevalence proportion	Number of cases	Prevalence proportion	Number of cases	Prevalence proportion
**Age ≥60 years**								
Men	19 388 180		19 780 650	··	20 501 800	··	19 896 410	··
Mild cognitive impairment	2 099 367 (2 059 812–2 141 539)	10·83% (10·62–11·05)	1 951 986 (1 905 139–1 996 513)	9·87% (9·63–10·09)	1 712 136 (1 679 667–1 740 805)	8·35% (8·19–8·49)	1 512 801 (1 484 225–1 542 434)	7·60% (7·46–7·75)
Dementia	1 574 037 (1 558 002–1 589 644)	8·12% (8·04–8·20)	1 701 501 (1 688 133–1 721 208)	8·60% (8·53–8·70)	1 512 435 (1 501 547–1 525 162)	7·38% (7·32–7·44)	1 176 225 (1 167 746–1 187 434)	5·91% (5·87–5·97)
Frailty	1 397 898 (1 396 632–1 400 103)	7·21% (7·20–7·22)	1 610 214 (1 608 468–1 612 455)	8·14% (8·13–8·15)	1 743 082 (1 741 000–1 744 713)	8·50% (8·49–8·51)	1 721 806 (1 719 608–1 725 415)	8·65% (8·64–8·67)
Dementia and frailty	419 282 (415 982–422 267)	2·16% (2·15–2·18)	494 973 (491 168–498 058)	2·50% (2·48–2·52)	483 458 (481 124–486 785)	2·36% (2·35–2·37)	402 453 (400 113–406 704)	2·02% (2·01–2·04)
Women	24 105 400	··	24 762 430	··	25 492 570	··	24 599 120	··
Mild cognitive impairment	2 510 807 (2 460 312–2 560 955)	10·42% (10·21–10·62)	2 719 972 (2 663 206–2 780 339)	10·98% (10·76–11·23)	2 714 563 (2 672 261–2 762 508)	10·65% (10·48–10·83)	2 353 775 (2 327 819–2 376 789)	9·57% (9·46–9·66)
Dementia	3 528 476 (3 517 009–3 542 491)	14·64% (14·59–14·70)	3 328 257 (3 316 729–3 343 257)	13·44% (13·39–13·50)	3 389 978 (3 376 754–3 402 069)	13·30% (13·25–13·34)	3 469 545 (3 459 747–3 482 466)	14·11% (14·06–14·15)
Frailty	2 736 493 (2 735 046–2 738 454)	11·35% (11·35–11·36)	3 159 041 (3 157 211–3 163 165)	12·76% (12·75–12·77)	3 462 453 (3 459 182–3 464 991)	13·58% (13·57–13·59)	3 514 460 (3 511 671–3 516 837)	14·29% (14·28–14·30)
Dementia and frailty	1 326 273 (1 322 800–1 329 978)	5·50% (5·49–5·52)	1 385 076 (1 381 675–1 387 750)	5·59% (5·58–5·60)	1 412 591 (1 410 049–1 415 646)	5·54% (5·53–5·55)	1 508 757 (1 505 482–1 512 162)	6·13% (6·12–6·15)
**Age 60–74 years**								
Men	12 495 730	··	10 983 780	··	11 737 240	··	11 556 310	··
Mild cognitive impairment	647 838 (623 913–669 322)	5·18% (4·99–5·36)	379 758 (365 668–393 103)	3·46% (3·33–3·58)	304 765 (294 038–318 124)	2·60% (2·51–2·71)	323 306 (312 044–335 989)	2·80% (2·70–2·91)
Dementia	241 230 (234 223–251 722)	1·93% (1·87–2·01)	120 409 (117 612–128 125)	1·10% (1·07–1·17)	73 529 (71 206–76 722)	0·63% (0·61–0·65)	80 786 (77 952–84 854)	0·70% (0·67–0·73)
Frailty	253 188 (252 590–254 024)	2·03% (2·02–2·03)	209 534 (208 508–210 371)	1·91% (1·90–1·92)	201 042 (200 269–202 084)	1·71% (1·71–1·72)	223 752 (222 768–224 374)	1·94% (1·93–1·94)
Dementia and frailty	22 988 (22 363–23 514)	0·18% (0·18–0·19)	12 625 (12 309–13 056)	0·11% (0·11–0·12)	7 443 (7 208–7 766)	0·06% (0·06–0·07)	8 511 (8 208–8 835)	0·07% (0·07–0·08)
Women	13 374 790	··	11 580 100	··	12 156 230	··	11 999 820	··
Mild cognitive impairment	652 112 (627 822–671 562)	4·88% (4·69–5·02)	575 006 (562 610–586 196)	4·97% (4·86–5·06)	576 853 (564 258–586 455)	4·75% (4·64–4·82)	588 701 (578 383–599 312)	4·91% (4·82–4·99)
Dementia	540 643 (533 481–550 283)	4·04% (3·99–4·11)	401 484 (394 366–407 183)	3·47% (3·41–3·52)	357 065 (351 389–364 288)	2·94% (2·89–2·100)	370 194 (363 351–378 297)	3·09% (3·03–3·15)
Frailty	256 670 (255 959–257 469)	1·92% (1·91–1·93)	225 264 (224 424–225 787)	1·95% (1·94–1·95)	202 606 (202 108–203 363)	1·67% (1·66–1·67)	219 516 (218 784–220 270)	1·83% (1·82–1·84)
Dementia and frailty	56 820 (55 923–57 626)	0·42% (0·42–0·43)	37 988 (37 091–38 776)	0·33% (0·32–0·33)	29 316 (28 786–29 932)	0·24% (0·24–0·25)	31 299 (30 685–31 974)	0·26% (0·26–0·27)
**Age ≥75 years**								
Men	6 892 449	··	8 797 099	··	8 764 496	··	8 340 157	··
Mild cognitive impairment	1 452 816 (1 421 541–1 485 732)	21·08% (20·62–21·56)	1 571 291 (1 530 316–1 614 157)	17·86% (17·40–18·35)	1 404 037 (1 381 904–1 436 634)	16·02% (15·77–16·39)	1 190 512 (1 162 623–1 210 344)	14·28% (13·94–14·51)
Dementia	1 330 830 (1 321 801–1 346 559)	19·31% (19·18–19·54)	1 579 892 (1 562 960–1 595 405)	17·96% (17·76–18·14)	1 438 631 (1 426 642–1 450 504)	16·41% (16·28–16·55)	1 094 243 (1 086 234–1 106 091)	13·12% (13·02–13·27)
Frailty	1 144 626 (1 143 366–1 146 433)	16·61% (16·59–16·63)	1 400 766 (1 398 867–1 402 590)	15·92% (15·90–15·94)	1 542 012 (1 540 086–1 543 719)	17·59% (17·58–17·61)	1 498 174 (1 496 468–1 501 385)	17·97% (17·94–17·99)
Dementia and frailty	396 540 (392 915–398 900)	5·75% (5·70–5·79)	482 238 (478 802–485 397)	5·48% (5·44–5·52)	476 089 (473 664–479 254)	5·43% (5·40–5·47)	393 916 (391 690–398 080)	4·72% (4·70–4·77)
Women	10 730 610	··	13 182 410	··	13 336 360	··	12 599 170	··
Mild cognitive impairment	1 862 290 (1 813 325–1 917 555)	17·35% (16·90–17·87)	2 146 789 (2 095 730–2 206 690)	16·29% (15·90–16·74)	2 136 943 (2 097 600–2 186 698)	16·02% (15·73–16·40)	1 763 372 (1 740 182–1 787 010)	14·00% (13·81–14·18)
Dementia	2 987 831 (2 977 104–2 996 943)	27·84% (27·74–27·93)	2 926 058 (2 914 632–2 941 153)	22·20% (22·11–22·31)	3 032 892 (3 020 980–3 047 625)	22·74% (22·65–22·85)	3 099 972 (3 092 386–3 108 864)	24·61% (24·55–24·67)
Frailty	2 479 944 (2 478 052–2 481 689)	23·11% (23·09–23·13)	2 933 980 (2 932 228–2 937 584)	22·26% (22·24–22·28)	3 259 602 (3 256 768–3 262 232)	24·44% (24·42–24·46)	3 294 875 (3 292 543–3 297 457)	26·15% (26·14–26·17)
Dementia and frailty	1 269 639 (1 266 877–1 273 129)	11·83% (11·81–11·86)	1 346 920 (1 343 984–1 349 643)	10·22% (10·20–10·24)	1 383 218 (1 380 498–1 386 477)	10·37% (10·35–10·40)	1 477 213 (1 474 770–1 480 889)	11·73% (11·70–11·75)

Data in parentheses are the 5th to 95th percentile range from 50 iterations.

**Table 2:** Projected costs for health care and formal long-term care per capita for the population aged 60 years and older, by sex and educational attainment

	Men		Women	
	Less than high school education	College education and higher	Less than high school education	College education and higher
**Year 2016**				
Total population				
Formal long-term care costs	$3569 (3568–3571)	$1825 (1823–1826)	$5517 (5515–5519)	$1974 (1972–1976)
Health-care costs	$4264 (4263–4266)	$4101 (4100–4102)	$4175 (4175–4176)	$2992 (2991–2993)
Mean number of comorbidities in population aged ≥60 years per capita	1·65	1·65	1·52	1·35
Without dementia or frailty				
Formal long-term care costs	$1407 (1392–1423)	$1428 (1425–1431)	$441 (438–444)	$752 (741–766)
Health-care costs	$3762 (3757–3768)	$3982 (3981–3983)	$3238 (3231–3243)	$2779 (2777–2781)
Mean number of comorbidities in population aged ≥60 years per capita	1·48	1·61	1·41	1·32
With dementia or frailty, or both				
Formal long-term care costs	$8950 (8882–9033)	$9134 (9093–9167)	$15 803 (15 753–15 840)	$15 880 (15 803–15 935)
Health-care costs	$5573 (5550–5599)	$6325 (6308–6343)	$6094 (6081–6109)	$5422 (5393–5449)
Mean number of comorbidities in population aged ≥60 years per capita	2·06	2·25	1·74	1·80
**Year 2043**				
Total population				
Formal long-term care costs	$4378 (4367–4385)	$2240 (2236–2243)	$8687 (8673–8701)	$3290 (3286–3293)
Health-care costs	$4337 (4333–4341)	$4281 (4280–4283)	$5140 (5135–5146)	$3237 (3236–3239)
Mean number of comorbidities in population aged ≥60 years per capita	1·73	1·80	1·62	1·38
Without dementia or frailty				
Formal long-term care costs	$1724 (1701–1746)	$1701 (1699–1704)	$823 (813–831)	$1140 (1125–1151)
Health-care costs	$3584 (3578–3590)	$4137 (4135–4139)	$3702 (3693–3713)	$2912 (2911–2914)
Mean number of comorbidities in population aged ≥60 years per capita	1·42	1·75	1·46	1·30
With dementia or frailty, or both				
Formal long-term care costs	$8849 (8786–8903)	$7904 (7887–7926)	$16 275 (16 236–16 314)	$15 717 (15 682–15 768)
Health-care costs	$5872 (5846–5895)	$6172 (6163–6182)	$6693 (6679–6707)	$5435 (5423–5447)
Mean number of comorbidities in population aged ≥60 years per capita	2·28	2·44	1·79	1·84

Data in parentheses are the 5th to 95th percentile range from 50 iterations. Annual cost estimations are in US$ at 2016 value. For mean number of comorbidities, uncertainty ranges were obtained but the ranges were too narrow to present here to a sufficient level of accuracy.

## Research in context

### Evidence before this study

We searched PubMed and Google on January 1–15, 2022, for academic papers and institutional reports published in English since database inception that forecast future disabilities related to cognitive impairment and physical frailty and their economic impact. Publications in Japanese were additionally searched. The search strategy included the terms “dementia”, “frailty”, “prevalence”, “cost of illness”, “burden of disease”, “forecasting”, and “computer simulation”. We identified ten simulation models developed for forecasting prevalence of dementia in England, the USA, Canada, Australia, Spain, Ireland, and globally. We did not find any studies explicitly including frailty, although two models considered the status of dependency or difficulties engaging in activities of daily life. Three models assessed the population impact of dementia on disability-free life expectancy, and five models assessed the estimated health-care cost. Three models involved microsimulations that considered multi-morbidity conditions of individual older people in the forecasting estimation, three models incorporated cardiovascular risk factors, and three models adopted state transition for the natural course of dementia progression. A study based on the Global Burden of Diseases, Injuries, and Risk Factors Study estimated the trend of prevalence of dementia regressed on the summary score of risk factors and educational attainment trend at the country level.

### Added value of this study

To our knowledge, this is the first microsimulation modelling study to simultaneously consider the population impacts of frailty and dementia in an ageing population. We projected that in the next two decades, when the older population in Japan will be at its largest, increased life expectancy for people aged 65 years and over will feature more years free of dementia, but fewer years without frailty than in 2016. A larger proportion of women were affected by both conditions. We predict that the prevalence of dementia will be greater in those with lower compared to higher educational attainment, and that this disparity will grow over time. The expected cost of health care and formal long-term care for those with dementia is expected to remain similar to that in 2016, whereas the cost associated with frailty care is expected to increase substantially.

### Implications of all the available evidence

Our results highlight a double burden of dementia and frailty among women aged 75 years and older and those with lower education, who will require complex care services at a higher cost per capita. Sex and educational disparities in comorbid dementia and frailty among the older population will be especially important to consider in the public health policy agenda for population ageing.
