# Supplementary material for: Projecting prevalence of frailty and dementia and the economic cost of care in Japan from 2016 to 2043: a microsimulation modelling study
Source: Lancet Public Health. Author manuscript; Available in PMC 2022 May 11. (PMC9094718; doi:10.1016/S2468-2667(22)00044-5)
Supplement: 1 [file NIHMS1801900-supplement-1.pdf]

THE LANCET
Public Health

Supplementary appendix

This appendix formed part of the original submission and has been peer reviewed. We post it as supplied by the authors.

Supplement to: Kasajima M, Eggleston K, Kusaka S, et al. Projecting prevalence of frailty and dementia and the economic cost of care in Japan from 2016 to 2043: a microsimulation modelling study. *Lancet Public Health* 2022; **7:** e458–68.

List of appendices

Literature review results for Research in Context Page 3

Appendix Table 1. Literature review of simulation studies forecasting dementia

Appendix Technical Note 1. Data sources and estimation strategy for the microsimulation model (Japanese Future Elderly Model ver. 4 [JFEMv4]) Page 8

Supplementary Table 1. Variable definitions and data sources

Appendix Technical Note 2. Estimation of cognitive impairment and frailty prevalence by probabilistic extrapolation Page 13

Supplementary Table 2. Multivariate probit estimators and standard errors (in parentheses) for calculating probability of cognitive impairment

Supplementary Table 3. Coefficients of logistic regression and standard errors (in parentheses) for calculating probability of frailty

Appendix Technical Note 3. Estimation of economic costs for care Page 19

Supplementary Table 4. OLS estimates for extrapolation of monthly healthcare costs in USD

Supplementary Table 5. Validation results of annual healthcare costs for 2016 in billion USD

Supplementary Table 6. Mean monthly utilisation of formal long-term care by age-sex-dependency strata and by service type (in USD)

Supplementary Table 7. Validation of the estimation of the number of formal long-term care service users and costs as of 2016 by referring to the government report

Appendix Table 2.

Validation of dementia/MCI prevalence estimation with reference to Asada (2013) and Ninomiya (2014) Page 26

Appendix Table 3.

Validation of frailty prevalence estimation with reference to Murayama et al. (2020) Page 27

Supplementary table for Appendix Tables 2 and 3.

Calibrated number of dementia, mild cognitive impairment (MCI), and Frailty in 2013 JFEM population for validation

Appendix Table 4. Page 29

Projected prevalence of dementia, mild cognitive impairment, and frailty by educational strata in 2016 and 2043

Supplementary tables for Appendix Table 4.

Projected number of dementia, mild cognitive impairment, and frailty by educational strata in 2016

Projected number of dementia, mild cognitive impairment, and frailty by educational strata in 2043

Appendix Table 5. Page 32

Total life expectancy and life expectancy with dementia, frailty, and both by education and sex strata

Appendix Figure 1. Page 33

Expected life expectancy at age 65 years free of (A) dementia and (B) frailty, 2016–2043, by a static assumption model with reference to existing life expectancy estimation by the National Institute of Population and Social Security, Japan

Appendix Figure 2 and Appendix Table 6. Page 34

Estimated education strata with reference to Barro-Lee data

Appendix Figure 3. Page 36

Population pyramid by cognitive functional status (A) and frailty status (B) corresponding to Table 1

Literature review results for Research in Context

As of January 2022, we reviewed existing computer simulation models that forecast future prevalence of dementia and/or frailty, and studies projecting their expected healthcare and social costs. We searched PubMed using the following search strategy, and complemented this search using Google to include published reports and grey papers. We also manually searched existing literature and gray papers published in Japanese.

PubMed search strategy

1. (frailty [MeSH] OR dementia [MeSH]) AND ("computer simulation"[MeSH] or "forecasting"[MeSH]) and ("cost of illness"[MeSH] or "burden of disease"[MeSH])
2. (frailty [MeSH] OR dementia [MeSH]) AND ("computer simulation"[MeSH] or "forecasting"[MeSH]) and "prevalence"[MeSH]

For dementia conditions, we identified ten streams of simulation studies that forecast future prevalence/incidence of dementia in the UK, the USA, Spain, Canada, Australia, and Ireland (Appendix Table 1).[1-15] The available worldwide reports and grey papers relied on macro projection with statistical assumption using the estimated future population component and currently estimated strata-specific prevalence of the target condition, which are excluded from the survey. There were two streams of studies (PACSim[1,2] and FEM-based[5,6]) that included multi-comorbidity conditions, including activities of daily life and/or dependency in daily life, in the forecasting simulation. Two other studies (IMPACT-BAM[3,4] and a Spanish study[11]) included cardiovascular risk factors in the dementia prevalence forecast. Baseline estimation of dementia conditions was based on empirical cognitive function assessment in social surveys in three study streams (PACSim, IMPACT-BAM, and FEM). These studies assess the population impact of dementia on life expectancy.[1,3,6] A recent GBD-based study estimated the trend of dementia prevalence regressed on the summary score of risk factors as well as educational attainment trend by country levels.[15]

We did not identify any studies that explicitly forecast the future frailty trend, presumably because of the current lack of consensus on the measurement of frailty, and limited prevalence data on the condition. PACSim forecast the prevalence of elderly people with dependency needs,[1] and FEM forecast those having difficulties with daily life activities.[5,6]

References for Research in Context

1. Kingston A, Comas-Herrera A, Jagger C; MODEM project. Forecasting the care needs of the older population in England over the next 20 years: estimates from the Population Ageing and Care Simulation (PACSim) modelling study. *Lancet Public Health*. 2018;**3**:e447–e455. doi: 10.1016/S2468-2667(18)30118-X.
2. Wittenberg R, Hu B, Jagger C, et al. Projections of care for older people with dementia in England: 2015 to 2040. *Age Ageing*. 2020;**49**:264–269. doi: 10.1093/ageing/afz154.
3. Ahmadi-Abhari S, Guzman-Castillo M, Bandosz P, et al. Temporal trend in dementia incidence since 2002 and projections for prevalence in England and Wales to 2040: modelling study. *BMJ*. 2017;**358**:j2856. doi:

10.1136/bmj.j2856.

4. Guzman-Castillo M, Ahmadi-Abhari S, Bandosz P, et al. Forecasted trends in disability and life expectancy in England and Wales up to 2025: a modelling study. *Lancet Public Health*. 2017;**2**:e307–e313. doi: 10.1016/S2468-2667(17)30091-9.
5. Zissimopoulos J, Crimmins E, St Clair P. The Value of Delaying Alzheimer's Disease Onset. *Forum Health Econ Policy*. 2014; **18**:25–39. doi: 10.1515/fhep-2014-0013.
6. Zissimopoulos JM, Tysinger BC, St Clair PA, Crimmins EM. The Impact of Changes in Population Health and Mortality on Future Prevalence of Alzheimer's Disease and Other Dementias in the United States. *J Gerontol B Psychol Sci Soc Sci*. 2018;**73**:S38–S47. doi: 10.1093/geronb/gbx147.
7. Brookmeyer R, Abdalla N, Kawas CH, Corrada MM. Forecasting the prevalence of preclinical and clinical Alzheimer's disease in the United States. *Alzheimers Dement*. 2018;**14**:121–129. doi: 10.1016/j.jalz.2017.10.009.
8. Manuel DG, Garner R, Finès P, et al. Alzheimer's and other dementias in Canada, 2011 to 2031: a microsimulation Population Health Modeling (POHEM) study of projected prevalence, health burden, health services, and caregiving use. *Popul Health Metr*. 2016;**14**. doi: 10.1186/s12963-016-0107-z.
9. Vickland V, McDonnell G, Werner J, Draper B, Low LF, Brodaty H. In silico modeling systems: learning about the prevalence and dynamics of dementia through virtual experimentation. *Alzheimers Dement*. 2011;**7**:e77–83. doi: 10.1016/j.jalz.2010.11.011.
10. Vickland V, Werner J, Morris T, et al. Who pays and who benefits? How different models of shared responsibilities between formal and informal carers influence projections of costs of dementia management. *BMC Public Health*. 2011;**11**. doi: 10.1186/1471-2458-11-793.
11. Soto-Gordoa M, Arrospide A, Moreno-Izco F, Martínez-Lage P, Castilla I, Mar J. Projecting Burden of Dementia in Spain, 2010-2050: Impact of Modifying Risk Factors. *J Alzheimers Dis*. 2015;**48**:721–30. doi: 10.3233/JAD-150233.
12. Standfield LB, Comans T, Scuffham P. A simulation of dementia epidemiology and resource use in Australia. *Aust N Z J Public Health*. 2018;**42**:291–295. doi: 10.1111/1753-6405.12700.
13. Standfield L, Comans T, Scuffham PA. Simulation of health care and related costs in people with dementia in Australia. *Aust Health Rev*. 2019;**43**:531–539. doi: 10.1071/AH18022.
14. Pierse T, Keogh F, O'Neill S. Generating national projections of dementia cases for Ireland using a calibrated macro-simulation model. *BMJ Open*. 2020;**10**:e035463. doi: 10.1136/bmjopen-2019-035463.
15. GBD 2019 Dementia Forecasting Collaborators. Estimation of the global prevalence of dementia in 2019 and forecasted prevalence in 2050: an analysis for the Global Burden of Disease Study 2019. *Lancet Public Health* 2022. https://doi.org/10.1016/ S2468-2667(21)00249-8.

Appendix Table 1. Literature review of simulation studies forecasting dementia

Model	Reference	Population	Age	Time horizon for projection	Model variables	Outcomes: Prevalence of dementia	Outcomes: Life expectancy/ Disability-free life expectancy	Outcomes: Cost: Healthcare including long-term care	Outcomes: Informal cost
Population Ageing and Care Simulation (PACSim)	Kingston et al., 2018[1]; Wittenberg et al., 2020[2]	UK	65+	2015–2035	age, sex, coronary heart disease, stroke, hypertension, diabetes, arthritis, cancer, respiratory disease, depression, vision, hearing impairments	0	0	0	0
IMPACT-Better Ageing Model (IMPACT-BAM)	Ahmadi-Abhari S et al., 2017[3]; Guzman-Castillo M et al., 2017[4]	UK	65+	2010–2040	age, sex, cardiovascular comorbidity	0	0	X	X
Future Elderly Model (FEM)	Zissimopoulos et al., 2014[5], 2018[6]	USA	65+	2010–2040	age, sex, education, race, iADL, BM, smoking, comorbidity (diabetes, heart disease, hypertension, stroke, lung disease, cancer)	0	0	X	X

Forward calculation method (multi-state model based on National Institute on Ageing Alzheimer's Association Framework)	Brookmeyer et al., 2018[7]	USA	30+	2017–2060	age-sex-specific transition probability of multiple clinical stages of Alzheimer's disease	0	X	X	X
Population Health Modeling (POHEM)	Manuel et al., 2016[8]	Canada	40+	2011–2031	age, sex, health status (HUI), mortality rate	0	X	0	0
Dementia Prevalence Model	Vickland et al., 2011a[9], 2011b[10]	Australia	60+	2010–2040	Estimated dementia prevalence by severity, location, type of care	0	X	0	0
Discrete event model based on Cardiovascular Risk Factors, Ageing, and Incidence of Dementia Risk Score	Soto-Gordoa M et al., 2015[11]	Spain	65+	2010–2050	age, sex, cardiovascular risk factors	0	X	0	X
Discrete event model with dynamic queueing	Standfield et al., 2018[12], 2019[13]	Australia	50+	2011–2050	age-sex- specific incidence rate of dementia by referring to Fratiglioni et al., 2020 Neurology	0	X	0	X
Macrosimulation with a multi-state Markov illness-death model	Pierse et al., 2020[14]	Ireland	65+	2016–2036	Three statuses (no dementia, dementia, death) with transition probabilities by referring to existing literature	0	X	X	X

Macrosimulation with linear regression of logit-transformed dementia prevalence by country, augmented with random walk modelling of unexplained residuals	GBD Dementia Forecasting Collaborators[15]	Global	40+	2019–2050	Summary exposure value (SEV) to summarise exposure to risk factors (low physical activity, hypertension, alcohol use, air pollution) in addition to years of education	0	X	X	X

iADL = instrumental activities of daily living; BM = body mass; HUI = Health Utilities Index.

Appendix Technical Note 1. Data sources and estimation strategy for the microsimulation model (Japanese Future Elderly Model ver. 4 [JFEMv4])

Chen et al. (2016) developed the first version of the Japanese Future Elderly Model (JFEM) using a panel dataset derived from the Japanese Study of Aging and Retirement (JSTAR).[16] Owing to the limited availability of data in the older-old aged strata, the estimation of physical function decline was possibly overestimated and unstable. Kasajima et al. (2020) overcame this limitation using nationally representative repeated cross-sectional data covering a wider range of age strata and by introducing the multi-state transition model.[17] The current model, JFEM ver. 4, further integrated education strata into Kasajima et al.'s model by referring to the education-related mortality risk ratio estimated in Kasajima and Hashimoto (2020).[18]

The JFEM model ver. 4 relied on 2010–2016 data from the Comprehensive Survey of People's Living Conditions, a nationally representative household survey of health conditions conducted every 3 years by the Ministry of Health, Labour and Welfare to calculate the conditional incidence probabilities of 11 chronic disease conditions (diabetes, coronary heart disease, stroke, hypertension, hyperlipidaemia, cancer, all respiratory diseases, joint disorders, eye diseases, kidney disorders, other chronic conditions), psychological distress/depression, two functional statuses (limitations in activities of daily living (ADL) and instrumental activities of daily living (iADL)), and subjective poor health status using a multi-state life table approach (Kasajima et al. 2020).[17] In brief, we estimated the number of incident cases within the cohort based on changes in disease prevalence between the periods between time t and $t + 1$, after removing the number of deaths. Namely, our model assumed, for two arbitrary diseases d_i and d_j $(i,j=1,\ldots,14;\ i\neq j)$, the following equation holds:

$$survivor_{(d_i,d_j)}(t+1) = survivor_{(d_i,d_j)}(t) - \int_t^{t+1} mortality_{(d_i,d_j)}(\tau)\, d\tau + \int_t^{t+1} incidence_{(d_i,d_j)}(\tau)\, d\tau.$$

Our model assumed Granger causality because our aim was to predict future health states, not to identify causal pathways. All chronic conditions were assumed to be absorbing states (no recovery). Our model limited the population at risk for a disease-specific death to those who had that disease. Age-sex-condition-specific case fatalities for corresponding years were estimated using vital statistics microdata under additive assumptions (i.e., case fatality of a comorbidity condition is assumed to be additive when calculating probabilities of corresponding comorbidity death rates). We estimated simulation parameters using Python 3.7.7.

In this study, age-sex-disease-specific case fatalities and all-cause mortality were calculated for three educational levels (lower than high school, high school, and university including junior college and vocational school) using the census-mortality linkage method (Kasajima and Hashimoto 2020). We required this process because death records in Japan do not contain educational attainments or personal identification numbers. We calculated the incidence rate ratio of mortality relative to primary education using Poisson regression between 2000 and 2010 for each birth year cohort in 3-year increments, and then extended the ratios to 2015. We estimated the sex, birth year cohort, and education-specific baseline population as of 2016 based on the 2000 census population because the 2010 census had a lower response rate and a higher proportion of missing educational information. A population

aged 80 years or older was estimated using the survival ratio method and extinct cohort method, following the Human Mortality Database protocol (available at https://www.mortality.org/).

We applied a census-mortality linkage approach to generate a future population of incoming cohorts aged 60–62 years every 3 years. For each educational group, we generated the number of death exits from the 2000 census population based on age-sex-education-specific all-cause mortality rates until the cohorts reached an age of 60 years, at which point they reached the age for microsimulation cohort entry. We started our simulation with a population aged 60 years or older and their health conditions as of June 2016 as the baseline. Owing to deterministic linkage allowing 1:m matching, the education-related mortality risk ratio may be underestimated in urban settings for the younger population, in which a larger number of "m" were matched to one death record, which may have led us to underestimate the education-related disparity in future health projection.

For future projections, the estimated condition-specific incidence and case fatalities in the most recent year (2015) were adopted in an estimated transition probability matrix for the first-order Markov model. We used a half-year cycle rather than a 1-year cycle in the Markov model because some disease conditions, especially cancer, have a turnover shorter than one year. This precludes the estimation of transition probability owing to uncounted death exits that may have exceeded new incidences in the middle of the cycle length if we had used a 1-year cycle. Therefore, we used a half-year cycle to obtain a balance between exit and entry.

As described above, our modelling of state transition was deterministic rather than stochastic, in that we deterministically obtained state-transition probabilities for projection estimation of future prevalence of states based on available epidemiological data. Instead, we stochastically prepared the baseline population by implementing 50 iterations of bootstrap simulation, and obtained the Monte Carlo error and 5th–95th percentile ranges. For each iteration, we refreshed the individual's probabilities of cognitively normal and frailty conditions each time.

Finally, it should be noted that the Comprehensive Survey of People's Living Conditions is a household-based community survey that excludes institutionalised/hospitalised individuals, which may have led to underestimation of morbidity prevalence. We calibrated the number to match existing morbidity data available in the Patient Survey. Technical details are available in Appendix A. Technical Document A1.3. Inflation of numbers of cancer prevalence in Kasajima et al. (2020).

Reference

16. Chen BK, Jalal H, Hashimoto H, Suen SC, Eggleston K, Hurley M, Schoemaker L, Bhattacharya J. Forecasting Trends in Disability in a Super-Aging Society: Adapting the Future Elderly Model to Japan. J Econ Ageing. 2016 Dec;8:42-51. doi: 10.1016/j.jeoa.2016.06.001.

17. Kasajima M, Hashimoto H, Suen SC, Chen B, Jalal H, Eggleston K, Bhattacharya J. Future projection of the health and functional status of older people in Japan: A multistate transition microsimulation model with repeated cross-sectional data. *Health Econ* 2020; **10**. doi: 10.1002/hec.3986.

18. Kasajima M, Hashimoto H. Social policies and change in education-related disparities in mortality in Japan, 2000–2010. *SSM Popul Health* 2020; **12**: 100692. doi: 10.1016/j.ssmph.2020.100692.

Supplementary Table 1. Variable definitions and data sources

Category	Components	Definition	Data source
Health	Diagnostic statuses of chronic diseases	Diabetes, coronary heart disease, stroke, hypertension, hyperlipidaemia, cancer, all respiratory diseases, joint disorders, eye diseases, kidney disorders, and other*	Comprehensive Survey of People's Living Conditions 2010, 2013, and 2016
	Psychological distress	Cut-off at 5 points on Kessler-6 scale	
	Subjective health	Poor or very poor	
	Dysfunctions in activities of daily living	Limitations in at least one of the following basic activities: independently getting out of bed, bathing, dressing, and eating	
	Dysfunction in mobility	Needs of care attention or assistance when going out	
Mortality	Cause-specific death based on the International Classification of Diseases 10th Revision (ICD-10)	Diabetes (E10–E14), coronary heart disease (I20–I25), stroke (I60–I69), hypertension (I10, I11, I12, I13, I15), hyperlipidaemia (E78), cancer (C00–C97), all respiratory diseases (J10–J22, J40–J47, J60–J70, J80–J84, J99, A15–A16), joint disorders (M05–M08, M10–M14, M15–M19, M40–M54), eye diseases (H25–H28, H30–H36, H40–H42), kidney disorders (N00–N07, N10–N15, N17–N19), others (I00–I09, I26–I52, K00–K99, B15–B19, N40), mental disorders (F20–F48, X60–X84)	Vital statistics 2010.1.1. to 2015.12.31
Population	Age-sex-education-specific population in 2015	Education-specific population estimated by 2010 census population and education disparity by age, sex	Population census 2010
Education gradients	Educational disparity in 2015 in base mortality and disease-specific mortality	Extension on the line through 2000 and 2010 educational disparities	Census-mortality linkage data of 2000 and 2010
Cognitive assessment	Amnestic mild cognitive impairment	Immediate word recall (cut-off at 3), delayed word recall (cut-off at 2), and serial-7 examinations (cut-off at 2): accompanied by at least one out of seven instrumental activities of daily living (using transportation, grocery shopping, preparing hot meals, paying bills, making deposits and withdrawals, using phones, and taking medication)	Japanese Study of Aging and Retirement 2007 and 2009

Frailty assessment	Frailty status using a Japanese modification of the Cardiovascular Health Study criteria	Shrinking (lost ≥2 kg in 6 months), weakness (grip strength <28 kg in men or <18 kg in women), exhaustion, slowness (gait speed <1.0 m/s), and low activity (moderate/low levels of physical exercise)	Kashiwa study
Economic outcomes	Healthcare costs and utilisation	Monthly utilisation of healthcare by service type (inpatient, outpatient, and pharmaceutical services) for acute and chronic conditions	National Database of Health Insurance Claims (NDB)
	Long-term care costs and utilisation	Monthly utilisation of formal long-term care for home and community-based care, care at care homes, and chronic care hospital beds	National long-term care (LTC) insurance claims

*The “other” category included circulatory diseases other than coronary heart disease (e.g., heart failure), gastric diseases, and non-cancer prostatic conditions (e.g., hyperplasia).

Appendix Technical Note 2. Estimation of cognitive impairment and frailty prevalence by probabilistic extrapolation

We focused on frailty and dementia as a policy target for integrated formal medical and personal care, which is likely to require substantial resources given the aged population. We used mild cognitive impairment (MCI) as a precursor stage of dementia.

For dementia and MCI predictions, we created three binary outcome variables, defined as poor performance at immediate word recall (y_1), delayed word recall (y_2), and serial-7 examinations (y_3), accompanied by iADL limitations.

In the JSTAR battery for cognitive functional measurement we relied on in this estimation, the orientation to date and place, immediate and late recall of 10 nouns, and serial-7 examinations were used to enable comparison with its sister survey, the Health and Retirement Study (HRS). However, because the word recall response is susceptible to cultural and linguistic differences (Dodge, et al.2009),[19] the JSTAR team referred to an existing word recall battery in the Alzheimer's Disease Assessment Scale cognitive subscale (ADAS-COG) Japanese version-11, developed by Gondo et al. (2004),[20] the response set of which was later validated by Kureta et al. (2007).[21]

Although we acknowledge that the Mini-Mental State Examination (MMSE) is the most widely used screening test, the Japanese version of the MMSE has been recognised as having several pitfalls for cross-country comparative purpose until very recently (Sugishita, et al. 2018).[22] Therefore, the JSTAR team decided to prioritise comparability with the HRS battery, and did not adapt the MMSE items for use.

Test responses, including word recall and serial-7 examination responses, were used as a survey-based classification system of cognitive impairment in the HRS, known as the Langa–Weir approach (Langa, et al., 2016).[23] We followed this approach, assuming that MCI and dementia are on a unidimensional spectrum of cognitive impairment. However, we acknowledge that this issue is controversial and that some researchers consider MCI and dementia to be distinct clinical concepts.

The diagnostic performance of the classification system was discussed in Crimmins et al. (2011),[24] who found that simple use of word recall (initial and late) and serial-7 examinations had a precision rate of approximately 60%. This rate increased to approximately 80% when age, sex, educational attainment, and limitations in ADL and iADL were used in addition to the test response in the regression model.

With this caveat as per Crimmins et al. (2011),[24] we chose in the current simulation study to regress the test performance of word recall (initial and late) and serial-7 examinations on age, sex, comorbidity status, ADL limitations, psychological distress (or depression measured by the Kessler-6 scale and/or the Center for Epidemiologic Studies Depression Scale), and educational attainment to reduce the effect of test measurement

error. Because word recall and serial-7 examination performance failure were differently predicted by these regression variables, we chose to run multivariate seemingly related probit models to simultaneously regress three test outcomes on the same set of above-mentioned explanatory variables, while allowing intercorrelation of error terms in simultaneous equations (see Supplementary Table 2 for regression results). We obtained the probability of non-failure of all three tests based on the estimated joint distribution of failure probabilities of these tests. We used this obtained probability as an indicator of cognitive function; higher probability was associated with better cognitive function.

The predicted test failure probabilities were extrapolated on an individual basis in a virtual cohort of the future older Japanese population prepared on the simulation system, according to age, sex, educational attainment, comorbidities, psychological distress, and limitations in ADL.

Then, we set the threshold for estimated probability of having MCI or dementia by referring to existing prevalence reports of total MCI/dementia numbers in Japan as of 2012 (e.g., Asada, et al.2013 [25]and Ninomiya, et al. 2014[26]). We assumed that MCI and dementia were situated on a unidimensional spectrum of cognitive dysfunction, and that the estimated probability of cognitive test non-failure would be distributed from high to low across normal function, MCI, and dementia, in that order.

Finally, we compared our estimated age-strata-specific prevalence with the reported numbers in Ninomiya and Asada, to see if our estimation model could reproduce the age-sex-specific distribution of dementia prevalence in the real world (Appendix Table 2). We took this reproduced distribution as support data for the validity of our estimation of dementia prevalence.

[Formulation]

We calculated the joint probability corresponding to a cognitively normal condition with $(y_1 = 0, y_2 = 0, and\ y_3 = 0)$ using the multivariate probit model:

$$y_m^* = \beta'_m \mathrm{X} + \varepsilon_m \quad (m = 1,2,3),$$

$$y_m = \begin{cases} 1 & if\ y_m^* > 0 \\ 0 & otherwise. \end{cases}$$

The common predictor X contained age, educational attainment, comorbidity conditions, self-rated health, ADL disability, and psychological distress conditions. Supplementary Table 1 shows the coefficients of multivariate probit regression β_m $(m = 1,2,3)$ and the ρ matrix, which describe the correlation across the error terms, $\varepsilon_1, \varepsilon_2$, and ε_3. The probability of observing $(y_1 = 0, y_2 = 0, and\ y_3 = 0)$ is approximated by a product of three independent standard normal random variables and Cholesky decomposition of a ρ matrix, called the Geweke–Hajivassiliou–Keane (GHK) simulator. We ran 100 iterations of the GHK simulator to obtain a numerical approximation. For 50 iterations of the JFEM simulation, 5,000 iterations in the GHK simulator were implemented. We sequentially indicated the probability thresholds of dementia and MCI conditions from the lowest value of the

joint probability to meet the previously reported prevalence of dementia and MCI in Japan [Asada (2013)[25] and Ninomiya (2014)[26]]. The dementia probability thresholds were 0·6606126 for men and 0·7162467 for women. The MCI probability thresholds were 0·7738026 for men and 0·8303307 for women.

We used logistic regression to predict frailty conditions for comparison with previous studies on frailty risk factors. We regressed the frailty condition according to age, sex, and self-reported morbidity of diabetes, heart disease, stroke, hypertension, hyperlipidaemia, malignant neoplasm, chronic renal failure, and impaired mobility (Supplementary Table 3). Using regression coefficients and standard errors, we calculated the cumulative logistic distribution based on each individual's age, sex, education, and health condition. We assigned a probability threshold to match the prevalence reported by an external source (Murayama et al. 2020)[27]. The inverse probability thresholds of frailty conditions were 1·762711 for men and 2·387823 for women.

References

19. Dodge, H., Meguro, K., Ishii, H., Yamaguchi, S., Saxton, J., & Ganguli, M. (2009). Cross-cultural comparisons of the Mini-mental State Examination between Japanese and U.S. cohorts. *International Psychogeriatrics, 21*(1), 113-122. doi:10.1017/S1041610208007886
20. Gondo Y, Fushimi T, Sakuma N, Amano S, Tatsumi IF, Homma A. Revision of word recall and word recognition task in Japanese version of Alzheimer's Disease Assessment Scale cognitive subscale (ADAS-J cog.). Ronen Seishin Igaku Zasshi. 15(8) :965-975, 2004.
21. Kureta Y, Gondo Y, Inagaki H, Fushimi T, Sakuma N, Homma A. Reliability assessment of the Alzheimer's Disease Assessment Scale for Japanese-cognitive subscale (ADAS-Jcog.) Ronen Seishin Igaku Zasshi. 18(4): 417-425, 2007.
22. Sugishita M, Koshizuka Y, Sudou S, Sugishita K, Hemmi I, Karawasa H, Ihara M, Asada T, Mihara B. The validity and reliability of the Japanese version of the Mini-Mental State Examination (MMSE-J) with the original procedure of the attention and calculation task (2001). Jpn J Cog Neurosci. 20(2); 91-110, 2018.
23. Langa KM, Weir DR, Kabeto M, Sonnega A. Langa-Weir Classification of Cognitive Function (1995 Onward). Survey Research Center, Institute for Social Research, University of Michigan. March, 2020. Available at https://hrsdata.isr.umich.edu/sites/default/files/documentation/data-descriptions/Data_Description_Langa_Weir_Classifications2016.pdf
24. Crimmins, E.M., Kim, J.K., Langa, K.M., & Weir, D.R. (2011). Assessment of cognition using surveys and neuropsychological assessment: the health and retirement study and the aging, demographics, and memory study. *The Journals of Gerontology, Series B: Psychological Sciences and Social Sciences, 66B(S1)*, i162–i171, doi:10.1093/geronb/gbr048
25. Asada T. The prevalence of dementia and the measures to functional disabilities in the urban areas. The Ministry of Health, Labour and Welfare grant system. 2013. https://mhlw-grants.niph.go.jp/niph/search/NIDD00.do?resrchNum=201218011A (in Japanese) (accessed June 1, 2018).
26. Ninomiya T. The study of future estimation of elderly population with dementia in Japan. The Ministry of

Health, Labour and Welfare grant system. 2014. https://mhlw-grants.niph.go.jp/niph/search/NIDD00.do?resrchNum=201405037A (in Japanese) (accessed June 1, 2018).

27. Murayama H, Kobayashi E, Okamoto S, Fukaya T, Ishizaki T, Liang J, Shinkai S. National prevalence of frailty in the older Japanese population: findings from a nationally representative survey. *Arch Gerontol Geriatr* 2020; **91**: 104220. doi: 10.1016/j.archger.2020.104220.

Supplementary Table 2. Multivariate probit estimators and standard errors (in parentheses) for calculating probability of cognitive impairment

	Men (N = 2145)			Women (N = 2311)		
	**Immediate word recall**	**Delayed word recall**	**Serial-7**	**Immediate word recall**	**Delayed word recall**	**Serial-7**
**Age**	0·02(0·01) **	0·04(0·01) ***	0·02(0·01) ***	0·04(0·01) ***	0·02(0·01) *	0·01(0·01)
**High school education**	−0·31(0·12) **	−0·36(0·12) ***	−0·36(0·10) ***	−0·17(0·14)	−0·14(0·12)	−0·32(0·10) ***
**College education**	−0·78(0·21) ***	−0·82(0·21) ***	−0·73(0·15) ***	−0·29(0·20)	−0·25(0·16)	−0·33(0·14) **
**Diabetes**	0·53(0·14) ***	0·74(0·13) ***	0·36(0·12) ***	0·34(0·19) *	0·27(0·17)	0·25(0·15)
**Heart disease**	−0·05(0·16)	0·02(0·15)	−0·06(0·13)	−0·41(0·23) *	−0·21(0·19)	−0·16(0·16)
**Stroke**	0·51(0·20) **	0·46(0·21) **	0·37(0·18) **	0·54(0·24) **	0·46(0·23) **	0·75(0·19) ***
**Hypertension**	−0·01(0·12)	−0·09(0·12)	−0·03(0·10)	−0·09(0·14)	−0·01(0·12)	−0·10(0·10)
**Hyperlipidaemia**	−0·60(0·28) **	−0·38(0·23) *	−0·57(0·22) **	0·00(0·19)	0·03(0·16)	−0·27(0·15) *
**Cancer**	0·20(0·27)	−0·08(0·29)	−0·12(0·24)	−0·30(0·36)	0·08(0·24)	0·05(0·22)
**Respiratory**	0·30(0·27)	0·29(0·26)	−0·96(0·44) **	0·37(0·25)	0·10(0·25)	−0·14(0·24)
**Joint**	0·25(0·29)	0·43(0·27)	−0·27(0·32)	−0·23(0·23)	−0·28(0·22)	−0·11(0·17)
**Eye**	0·11(0·16)	0·13(0·16)	0·08(0·14)	−0·16(0·17)	−0·12(0·15)	0·06(0·12)
**Other**	−0·24(0·16)	0·04(0·14)	−0·07(0·12)	0·08(0·20)	0·15(0·17)	0·04(0·15)
**Poor subjective health**	0·19(0·18)	0·07(0·18)	0·20(0·16)	0·30(0·18) *	0·25(0·18)	0·19(0·15)
**At least 1 ADL limitation**	0·08(0·23)	−0·16(0·25)	0·42(0·17) **	0·82(0·17) ***	0·67(0·17) ***	0·87(0·14) ***
**Psychological distress**	0·01(0·14)	0·18(0·13)	0·11(0·11)	0·14(0·14)	−0·15(0·13)	−0·18(0·11)
**Intercept**	−3·40(0·71) ***	−4·25(0·73) ***	−2·96(0·56) ***	−4·48(0·79) ***	−2·99(0·65) ***	−2·14(0·54) ***
**rho21**		0·76(0·04) ***			0·74(0·04) ***	
**rho31**		0·62(0·05) ***			0·52(0·05) ***	
**rho32**		0·68(0·04) ***			0·58(0·04) ***	

Note: ***p<0·01, **p<0·05, and *p<0·1. The dementia probability thresholds were 0·6606126 for men and 0·7162467 for women. MCI probability thresholds were 0·7738026 for men and 0·8303307 for women. ADL = activities of daily living; MCI = mild cognitive impairment.

Supplementary Table 3. Coefficients of logistic regression and standard errors (in parentheses) for calculating probability of frailty

	Men (N = 1011)		Women (N = 1028)	
**Age**	0·15 (0·02)	***	0·17 (0·02)	***
**High school education**	−0·63 (0·39)		−0·13 (0·25)	
**College education**	−0·50 (0·37)		−0·54 (0·34)	*
**Diabetes**	0·03 (0·33)		0·54 (0·34)	
**Heart diseases**	0·33 (0·28)		0·13 (0·27)	
**Stroke**	0·65 (0·36)	*	0·21 (0·41)	
**Hypertension**	0·37 (0·26)		0·20 (0·22)	
**Hyperlipidaemia**	−0·07 (0·28)		0·05 (0·21)	
**Cancer**	−0·14 (0·31)		0·25 (0·31)	
**Kidney disorders**	−0·17 (1·13)		1·76 (0·96)	*
**Psychological distress**	0·60 (0·30)	**	1·46 (0·24)	***
**Impaired mobility**	2·71 (1·38)	*	2·62 (1·18)	**
**Intercept**	−13·47 (1·67)	***	−14·75 (1·49)	***

Note: ***p<0·01, ** p<0·05, and *p<0·1. The frailty thresholds were 1·762711 for men and 2·387823 for women.

Appendix Technical Note 3: Estimation of economic costs for care

Healthcare costs

We estimated healthcare costs of inpatient medical services and outpatient services, including prescriptions. We analysed a 3% sample of the national electronic claims data (approximately 200 million records of 5 million individuals) for the national health insurance from April 2013 to March 2016. The claims data included monthly utilisation of healthcare by service type, with information on patient demographics and diagnoses. We regressed the utilisation of medical services for men and women separately according to age, morbidity diagnoses of 11 diseases, and the number of comorbidities (Supplementary Table 4).

For each individual i, we extrapolated the expected monthly utilisation for inpatient medical services (Inp_cost_i) and outpatient care and prescriptions ($\mathrm{Outp\_cost}_i$) by

$$Inp\_cost_i = \beta_0 + \alpha \; age_i + BH_i + \gamma \, n(H_i) + \varepsilon_i \;,$$
$$Outp\_cost_i = \beta_0 + \alpha \; age_i + B_{initial}H_i^{initial} + B_{follow-up}H_i^{follow-up} + \varepsilon_i,$$

with health conditions, H_i, and the number of comorbidities, $n(H_i)$. For outpatient services, we specified the initial consultation, $H_i^{initial}$, if the individual, i, was newly diagnosed in the corresponding month; otherwise, the consultation was regarded as a follow-up consultation (namely, $H_i = H_i^{initial} + H_i^{follow-up}$). We fixed the age effect, α, after age 85 years to fit our cost estimation per capita to the official governmental report.

We did not estimate an individual patient's status for requiring outpatient or inpatient services or both; therefore, we estimated the propensity of receiving inpatient services (P_i^{inp}) by referring to the number of inpatient cases out of the total claims for each age-sex strata under the health condition, H_i. Then, we obtained the total medical cost for the month by aggregating the expected utilisation of the simulated population:

$$Total\ medical\ cost = \sum_i \; P_i^{inp} \times Inp\_cost_i + \sum_i \; (1 - P_i^{inp}) \times Outp\_cost_i.$$

The monthly estimation was multiplied by 6 months for the estimation period cycle, and was finally summed for the annual estimation.

Reliance on diagnostic codes presented in administrative data may lead to the problem of overcoding and consequent misclassification of the resource utilisation linked to disease-specific conditions. Although we acknowledge this problem, we assigned health conditions by simply referring to recorded diagnostic codes.

As a validity check, we applied the obtained regression coefficients to the prepared virtual cohort of the older Japanese population (aged >65 years) in the simulation to extrapolate estimated individual monthly healthcare utilisation. We compared the sum of the estimated individual utilisation with the government report of national medical expenditure, as presented in Supplementary Table 5. The estimated healthcare cost as of 2016 successfully replicated the reported value, which we believe at least partly supports the validity of our population-level estimation of healthcare costs.

Supplementary Table 4. OLS estimates for extrapolation of monthly healthcare costs in USD

		Inpatient medical services		Outpatient services and prescriptions	
		**Men**	**Women**	**Men**	**Women**
**Intercept (β_0)**		4,641	5,045	272	223
**Age (α)**		40	−32	−3	−2
**Comorbidity (matrix B)**	**Condition**				
**Diabetes**	**Initial**	−207	−113	57	49
	**Follow-up**			35	31
**Ischemic heart disease**	**Initial**	1,327	821	98	71
	**Follow-up**			58	34
**Stroke**	**Initial**	617	901	48	50
	**Follow-up**			13	17
**Hypertension**	**Initial**	−21	−43	36	32
	**Follow-up**			7	8
**Hyperlipidaemia**	**Initial**	1	−199	−38	−14
	**Follow-up**			−53	−30
**Cancer**	**Initial**	507	375	399	427
	**Follow-up**			174	162
**Respiratory disease**	**Initial**	252	−176	87	64
	**Follow-up**			32	31
**Joint disorder**	**Initial**	268	926	76	71
	**Follow-up**			42	45
**Eye disease**	**Initial**	−546	−857	143	111
	**Follow-up**			78	55
**Kidney disorder**	**Initial**	577	233	493	358
	**Follow-up**			942	700
**Other circulatory diseases, gastric diseases, and prostate disorder**	**Initial**	544	352	102	91
	**Follow-up**			51	54
**Number of comorbidities (γ)**					
1		736	691		
2		1,473	1,266		
3		1,764	1,450		
4		1,931	1,553		
5+		2,113	1,791		

Note: We converted JPY to USD at the exchange rate 1,000 JPY = 9·091 USD. OLS = ordinary least squares.

Supplementary Table 5. Validation results of annual healthcare costs for 2016 in billion USD

	JFEM estimation				Government annual report of 2016*			
**Age**	**Inpatient medical services**		**Outpatient services and prescriptions**		**Inpatient medical services**		**Outpatient services and prescriptions**	
**60–74 years**	Men	26	Men	21	Men	25	Men	22
	Women	18	Women	18	Women	17	Women	20
**75+ years**	Men	23	Men	17	Men	28	Men	17
	Women	37	Women	22	Women	38	Women	22

Note: We converted JPY to USD at the exchange rate 1,000 JPY = 9·091 USD. JFEM = Japanese Future Elderly Model.
*Ministry of Health, Labour and Welfare. Estimates of national medical care expenditure. [cited 2021 Aug 3]. Available from: https://www.mhlw.go.jp/english/database/db-hss/enmce.html

Formal care cost for social care

To calculate the formal care cost of social care provided under the public long-term care (LTC) insurance scheme, we analysed the nationwide administrative data for the LTC plan of June 2016 (4 million individuals), which holds information regarding beneficiaries' age, sex, eligibility levels reflecting dependency status, and monthly utilisation by service type (home/community-based vs. care home-based). We calculated the mean value of monthly utilisation ($U_{sex,age,dep}^{community}$, $U_{sex,age,dep}^{care\ home}$) by age-sex-dependency strata and by service type (Supplementary Table 6), and then multiplied the estimated prevalence number of older people ($N_{sex,age,dep}^{community}$, $N_{sex,age,dep}^{care\ home}$) by age-sex-dependency strata to obtain the total cost of social care. We did not estimate the place of service reception in our simulation model; therefore, we estimated the propensity of service type use by referring to the ratio of community-based service users to care home users as of 2016 in each segment of dependency status for each sex and age category.

$$\text{Total cost of social care} = \sum_{sex} \sum_{age} \sum_{dep=mild,high} \left(N_{sex,age,dep}^{community} \times U_{sex,age,dep}^{community} + N_{sex,age,dep}^{care\ home} \times U_{sex,age,dep}^{care\ home} \right).$$

We validated our estimation by comparison with the official government report of 2016. As of 2016, the Comprehensive Survey of People's Living Conditions by the Ministry of Health, Labour and Welfare reported that only 63% of elderly people (40% in their 60s to 90% in their 90s) who needed care for emerging ADL/IADL dysfunctions actually applied for a formal LTC service. Thus, we present estimated formal care costs based on the as-is scenario (63% of those in need will use formal care) for comparison with the number reported in the government report. We confirmed that our as-is scenario corresponds well to the publicly announced actual use of formal LTC services (Supplementary Table 7).

Scenario analysis for sensitivity check

As we anticipate that the household capacity for informal care provision will decline over time in Japan owing to an expected reduction in household size, it is unclear whether formal care use will increase to substitute for the decline in informal care provision, given the mixed empirical findings in the literature.

We acknowledge a study by Bonsang (2009) in J Health Econ, which used European panel data derived from the Survey of Health, Ageing, and Retirement in Europe (SHARE), and concluded that a substitutional association was observed only for mild care needs.[28] Evidence from the USA suggests a substitution of formal care for people with dementia with fewer family members availabile to supply informal care (Choi et al. 2021)[29], and that better access to formal care support reduced coresidence with adult children (Mommaerts 2018).[30] Courbage et al. used SHARE data in a European study and found mixed results; formal care was substituted for informal care in Spain, but complemented informal care in Italy.[31] More recently, Lin (2019) reported the situation in China, and concluded that formal care is complementary rather than substitutional for informal care provision.[32] This issue remains understudied in Japan. Recently, Miyawaki et al. (2020) reported that the reduced availability of formal care for milder care needs after public policy change leads to increased hours of informal care provision.[33] This suggests a substitutional relationship in the case of mild care needs; however, the reverse pattern (that a reduction in informal care leads to a substitutional increase in formal care use) remains to be tested.

Given the available evidence on this issue, we concluded that there is no firm empirical basis on which to make assumptions about the resource utilisation patterns for formal and informal care. We adopted a conservative position and decided to assume that the pattern will remain constant over the next two decades. This assumption may underestimate the use of formal care services and overestimate the cost of informal care if the substitutional association holds in the case of mild care needs.

We conducted a sensitivity analysis in which we relied on the substitutional elasticity between formal community-based home care and informal care demonstrated in Bonsang (2009)[28] (substitutional elasticity of −0·68, or 10% increase in informal care leads to 6·8% decrease in formal care use). Bonsang found that the substitution of informal care provision for nursing care was not significant. We referred to a government projection that mean household size would decrease from 2·33 in 2015 to 2·08 in 2040, and estimated that the capacity of informal care (household size minus 1) is expected to decrease by approximately 19% (= 1−(2·08−1)/(2·33−1)). If we apply this number to our estimated cost of formal community-based home care services, a 19% decrease in informal care cost (=103·3 billion USD) will be substituted by an increase in formal care cost by 13%, or 11·9 billion USD (=83 billion USD*0·13) as of 2043.

The total cost of informal and formal care as of 2043 was estimated as 464·2 billion USD. After the substitution, the cost will be 455·5 billion USD, or 98·1% of the original estimate.

References

28. Bonsang E. Does informal care from children to their elderly parents substitute for formal care in Europe? J Health Econ. 2009 Jan;28(1):143-54. doi: 10.1016/j.jhealeco.2008.09.002.
29. Choi H, Heisler M, Norton EC, Langa KM, Cho TC, Connell CM. Family Care Availability And Implications For Informal And Formal Care Used By Adults With Dementia In The US. Health Aff (Millwood). 2021 Sep;40(9):1359-1367. doi: 10.1377/hlthaff.2021.00280.
30. Mommaerts C. Are coresidence and nursing homes substitutes? Evidence from Medicaid spend-down provisions. J Health Econ. 2018 May;59:125-138. doi: 10.1016/j.jhealeco.2018.04.003.
31. Courbage C, Montoliu-Montes G, Wagner J. The effect of long-term care public benefits and insurance on informal care from outside the household: empirical evidence from Italy and Spain. Eur J Health Econ. 2020 Nov;21(8):1131-1147. doi: 10.1007/s10198-020-01215-7.
32. Lin, W. The relationship between formal and informal care among Chinese older adults: based on the 2014 CLHLS dataset. *BMC Health Serv Res* **19,** 323 (2019). https://doi.org/10.1186/s12913-019-4160-8
33. Miyawaki, A., Kobayashi, Y., Noguchi, H. *et al.* Effect of reduced formal care availability on formal/informal care patterns and caregiver health: a quasi-experimental study using the Japanese long-term care insurance reform. *BMC Geriatr* **20,** 207 (2020). https://doi.org/10.1186/s12877-020-01588-7

Supplementary Table 6. Mean monthly utilisation of formal long-term care by age-sex-dependency strata and by service type (in USD)

	Home- and community-based care				Care at care homes/chronic care beds			
	Men		Women		Men		Women	
**Age (years)**	**High**	**Mild**	**High**	**Mild**	**High**	**Mild**	**High**	**Mild**
**60–62**	1,433	538	1,375	459	2,663	1,956	2,719	1,862
**63–65**	1,439	525	1,374	449	2,601	1,924	2,657	1,899
**66–68**	1,409	508	1,382	433	2,585	1,891	2,624	1,796
**69–71**	1,388	500	1,379	430	2,575	1,943	2,624	1,809
**72–74**	1,359	505	1,384	432	2,556	1,889	2,618	1,859
**75–77**	1,336	495	1,409	439	2,571	1,892	2,626	1,859
**78–80**	1,325	494	1,431	454	2,576	1,844	2,637	1,868
**81–83**	1,332	501	1,474	474	2,582	1,826	2,652	1,880
**84–86**	1,364	510	1,508	511	2,577	1,814	2,651	1,900
**87–89**	1,407	536	1,537	552	2,580	1,814	2,645	1,942
**90–92**	1,444	567	1,543	594	2,564	1,840	2,639	1,954
**93–95**	1,484	601	1,548	634	2,555	1,853	2,631	2,000
**96–98**	1,516	645	1,564	665	2,569	1,934	2,632	2,025
**99–101**	1,557	704	1,589	707	2,555	1,848	2,633	2,062
**102–104**	1,603	718	1,636	718	2,537	1,981	2,649	2,086
**105+**	1,704	642	1,787	810	2,582	2,277	2,664	1,964

Note. High is an abbreviation for high dependency (with disability conditions categorised in the higher four levels for long-term care service use); Mild is an abbreviation for mild dependency (the lower three levels). We converted JPY to USD at the exchange rate 1,000 JPY = 9·091 USD.

Supplementary Table 7. Validation of the estimation of the number of formal long-term care service users and costs as of 2016 by referring to the government report

	Home- and community-based care		Care at care homes	
	**JFEM estimation**	**Government report****	**JFEM estimation**	**Government report****
**As-is scenario (63% formal LTC service usage)**				
**Number of formal LTC service users (in millions)**	3·7	3·3	1·4	1·3
**Social care cost (billion USD)**	47·0	44·5	44·0	39·1
**100% formal care usage scenario**				
**Estimated number of formal LTC service users based on needs status (in millions)**	5·9	NA	2·1	NA
**Expected social care cost (billion USD)**	74·5	NA	65·8	NA

Note: We converted JPY to USD at the exchange rate 1,000 JPY= 9·091 USD. JFEM = Japanese Future Elderly Model; LTC = long-term care.

**Ministry of Health, Labour and Welfare. Survey of Long-term Care Benefit Expenditures. [cited 2021 Aug 3]. Available from: https://www.mhlw.go.jp/english/database/db-hss/soltcbe.html.

Appendix Table 2. Validation of dementia/mild cognitive impairment (MCI) prevalence estimation with reference to Asada (2013) and Ninomiya (2014)

<table>
<tr><th colspan="3">Calibrated dementia prevalence in 2013 JFEM population</th><th colspan="3">Dementia prevalence in Ninomiya (2014)</th></tr>
<tr><th>Age (years)</th><th>Men</th><th>Women</th><th>Age (years)</th><th>Men</th><th>Women</th></tr>
<tr><td>**65–69**</td><td>1·68%</td><td>4·19%</td><td>**65–69**</td><td>1·94% (1·44%–2·61%)</td><td>2·42% (1·81%–3·25%)</td></tr>
<tr><td>**70–74**</td><td>4·74%</td><td>7·51%</td><td>**70–74**</td><td>4·30% (3·31%–5·59%)</td><td>5·38% (4·18%–6·93%)</td></tr>
<tr><td>**75–79**</td><td>11·32%</td><td>14·31%</td><td>**75–79**</td><td>9·55% (7·53%–12·12%)</td><td>11·95% (9·57%–14·91%)</td></tr>
<tr><td>**80–84**</td><td>19·87%</td><td>23·45%</td><td>**80–84**</td><td>21·21% (16·86%–26·68%)</td><td>26·52% (21·57%–32·61%)</td></tr>
<tr><td>**85–89**</td><td>28·26%</td><td>38·47%</td><td rowspan="4">**≥85**</td><td rowspan="4">47·09% (37·09%–59·77%)</td><td rowspan="4">58·88% (47·69%–72·69%)</td></tr>
<tr><td>**90–94**</td><td>49·09%</td><td>55·77%</td></tr>
<tr><td>**95–99**</td><td>66·51%</td><td>71·60%</td></tr>
<tr><td>**≥100**</td><td>84·19%</td><td>87·17%</td></tr>
<tr><th colspan="3">Calibrated MCI prevalence in 2013 JFEM population</th><th colspan="3">MCI prevalence in nine municipalities (N = 5,902) in Asada (2013)</th></tr>
<tr><th>Age (years)</th><th>Men</th><th>Women</th><th>Age (years)</th><th>Men</th><th>Women</th></tr>
<tr><td>**65–69**</td><td>6·47%</td><td>4·41%</td><td>**65–69**</td><td>7·8% (5·8%–9·7%)</td><td>5·9% (4·3%–7·5%)</td></tr>
<tr><td>**70–74**</td><td>9·61%</td><td>6·13%</td><td>**70–74**</td><td>10·8% (8·5%–13·1%)</td><td>8·7% (6·8%–10·6%)</td></tr>
<tr><td>**75–79**</td><td>15·32%</td><td>11·28%</td><td>**75–79**</td><td>13·8% (11·3%–16·3%)</td><td>12·2% (9·9%–14·4%)</td></tr>
<tr><td>**80–84**</td><td>23·00%</td><td>17·91%</td><td>**80–84**</td><td>21·6% (18·3%–25·0%)</td><td>15·1% (12·6%–17·6%)</td></tr>
<tr><td>**85–89**</td><td>30·74%</td><td>21·50%</td><td>**85–89**</td><td>16·2% (12·7%–19·8%)</td><td>14·4% (11·4%–17·3%)</td></tr>
<tr><td>**90–94**</td><td>27·82%</td><td>25·86%</td><td>**90–94**</td><td>18·5% (13·4%–23·7%)</td><td>9·9% (6·9%–12·9%)</td></tr>
<tr><td>**95–99**</td><td>20·11%</td><td>22·99%</td><td>**95–99**</td><td>12·1% (5·5%–18·6%)</td><td>7·1% (3·1%–11·1%)</td></tr>
<tr><td>**≥100**</td><td>8·08%</td><td>12·69%</td><td>**≥100**</td><td>19·5% (0·0%–52·6%)</td><td>0·0% (0·0%–0·0%)</td></tr>
</table>

JFEM = Japanese Future Elderly Model.

Appendix Table 3. Validation of frailty prevalence estimation with reference to Murayama et al (2020)

**Calibrated frailty prevalence in 2013 JFEM population**			**Frailty prevalence in Murayama et al. (2020)**		
**Age (years)**	**Men**	**Women**	**Age (years)**	**Men**	**Women**
**65–69**	1·7%	1·6%	**65–69**	2·1%	2·5%
**70–74**	4·2%	4·0%	**70–74**	4·7%	3·8%
**75–79**	9·1%	9·6%	**75–79**	6·4%	7·7%
**80–84**	17·0%	19·5%	**80–84**	16·5%	14·6%
**85–89**	27·6%	31·8%	**≥85**	26·4%	32·1%
**90–94**	38·9%	45·6%			
**95–99**	50·3%	58·5%			
**≥100**	56·9%	66·6%			

JFEM = Japanese Future Elderly Model.

Supplementary Table for Appendix Tables 2 and 3; Calibrated number of dementia, mild cognitive impairment (MCI), and Frailty in 2013 JFEM population for validation

2013 JFEM population			Dementia		MCI		Frailty	
**Age (years)**	**Men**	**Women**	**Men**	**Women**	**Men**	**Women**	**Men**	**Women**
**65–69**	4,246,251	4,516,691	71,391	189,120	274,658	199,137	73,937	71,949
**70–74**	3,610,682	4,108,816	171,055	308,594	347,112	251,790	152,307	165,908
**75–79**	2,835,671	3,547,069	320,921	507,755	434,333	400,004	257,204	339,182
**80–84**	1,954,798	2,969,438	388,354	696,400	449,626	531,710	332,680	579,504
**85–89**	1,060,697	2,020,286	299,702	777,209	326,041	434,335	292,319	643,116
**90–94**	333,126	1,029,825	163,545	574,300	92,683	266,309	129,570	469,205
**95–99**	74,452	344,509	49,520	246,674	14,974	79,206	37,470	201,635
**≥100**	8,487	38,488	7,145	33,550	686	4,886	4,830	25,634

JFEM = Japanese Future Elderly Model.

Appendix Table 4. Projected prevalence of dementia, mild cognitive impairment, and frailty by educational strata in 2016 and 2043

			2016		2043	
			**Less than high school (%)**	**College and over (%)**	**Less than high school (%)**	**College and over (%)**
**60+ all**	**Male**	**MCI***	24·05 (23·28–24·77)	1·37 (1·32–1·41)	20·63 (19·97–21·25)	2·31 (2·26–2·35)
		**Dementia**	22·54 (22·23–22·82)	0·35 (0·33–0·37)	30·11 (29·73–30·32)	0·78 (0·76–0·80)
		**Frailty**	12·12 (12·11–12·15)	4·95 (4·94–4·96)	16·62 (16·58–16·67)	7·40 (7·39–7·42)
		**Dementia + frailty**	6·03 (5·97–6·06)	0·15 (0·14–0·16)	10·81 (10·73–10·88)	0·35 (0·34–0·36)
	**Female**	**MCI**	18·97 (18·36–19·57)	5·37 (5·27–5·44)	20·95 (20·47–21·54)	6·82 (6·75–6·96)
		**Dementia**	25·26 (25·15–25·40)	5·31 (5·25–5·37)	38·13 (37·95–38·28)	8·65 (8·60–8·71)
		**Frailty**	17·73 (17·71–17·76)	4·29 (4·28–4·31)	30·46 (30·42–30·53)	8·09 (8·08–8·11)
		**Dementia + frailty**	9·95 (9·91–9·98)	1·53 (1·52–1·54)	19·79 (19·68–19·86)	2·69 (2·68–2·70)
**60–74**	**Male**	**MCI**	16·90 (15·99–17·47)	0·20 (0·17–0·23)	17·93 (16·98–18·59)	0·16 (0·14–0·18)
		**Dementia**	7·32 (7·06–7·75)	0·05 (0·04–0·06)	6·81 (6·58–7·25)	0·01 (0·01–0·01)
		**Frailty**	3·38 (3·37–3·40)	1·63 (1·62–1·64)	3·53 (3·50–3·56)	1·83 (1·82–1·84)
		**Dementia + frailty**	0·68 (0·66–0·70)	0·01 (0·01–0·01)	0·73 (0·71–0·76)	0·00 (0·00–0·00)
	**Female**	**MCI**	7·45 (6·98–8·09)	4·62 (4·52–4·78)	7·80 (7·30–8·23)	5·06 (4·91–5·17)
		**Dementia**	8·55 (8·46–8·66)	1·95 (1·86–2·02)	11·47 (11·35–11·59)	2·09 (2·0–2·22)
		**Frailty**	2·88 (2·86–2·90)	1·14 (1·13–1·15)	3·49 (3·46–3·53)	1·36 (1·35–1·37)
		**Dementia + frailty**	0·88 (0·87–0·89)	0·15 (0·14–0·16)	0·95 (0·93–0·97)	0·13 (0·12–0·14)
**75 and +**	**Male**	**MCI**	30·65 (29·77–31·90)	4·98 (4·84–5·15)	23·17 (22·61–24·16)	5·77 (5·64–5·90)
		**Dementia**	36·72 (36·31–37·17)	1·27 (1·19–1·33)	52·63 (52·01–52·99)	2·02 (1·99–2·06)
		**Frailty**	20·23 (20·21–20·28)	15·27 (15·23–15·31)	29·31 (29·23–29·37)	16·41 (16·38–16·44)
		**Dementia + frailty**	11·00 (10·89–11·06)	0·58 (0·54–0·62)	20·58 (20·44–20·69)	0·91 (0·89–0·92)
	**Female**	**MCI**	25·30 (24·29–26·17)	8·22 (7·97–8·43)	27·22 (26·61–27·86)	9·42 (9·26–9·64)
		**Dementia**	34·41 (34·20–34·58)	18·39 (18·27–18·55)	50·70 (50·43–50·94)	18·26 (18·15–18·43)
		**Frailty**	25·87 (25·83–25·90)	16·53 (16·46–16·57)	43·17 (43·12–43·25)	17·97 (17·95–18·00)
		**Dementia + frailty**	14·91 (14·85–14·97)	6·89 (6·84–6·93)	28·67 (28·53–28·76)	6·46 (6·43–6·48)

*MCI = mild cognitive impairment. 5th and 95th percentile ranges are provided in parentheses.

Supplementary tables for Appendix Table 4.

Projected number of dementia, mild cognitive impairment (MCI), and frailty by educational strata in 2016

		Year 2016	Less than high school	5th-95th percentile range	College and over	5th-95th percentile range
**60+ all**	**Male**	**N**	5,367,950		5,039,513	
		**MCI**	1,291,084	(1,249,849-1,329,786)	68,949	(66,725-71,053)
		**Dementia**	1,210,074	(1,193,320-1,224,797)	17,506	(16,642-18,493)
		**Frailty**	650,671	(649,813-652,318)	249,584	(249,072-250,109)
		**Dementia + frailty**	323,942	(320,551-325,500)	7,583	(7,056-8,092)
	**Female**	**N**	7,915,682		3,650,107	
		**MCI**	1,501,491	(1,453,162-1,549,083)	195,866	(192,532-198,479)
		**Dementia**	1,999,558	(1,990,783-2,010,775)	193,967	(191,581-195,992)
		**Frailty**	1,403,811	(1,401,924-1,405,442)	156,674	(156,285-157,155)
		**Dementia + frailty**	787,529	(784,458-790,242)	55,869	(55,479-56,125)
**60-74**	**Male**	**N**	2,582,625		3,812,666	
		**MCI**	436,362	(412,968-451,139)	7,599	(6,659-8,621)
		**Dementia**	189,048	(182,216-200,088)	2,054	(1,664-2,364)
		**Frailty**	87,251	(86,906-87,749)	62,228	(61,893-62,478)
		**Dementia + frailty**	17,518	(17,005-17,957)	462	(390-563)
	**Female**	**N**	2,800,737		2,902,130	
		**MCI**	208,576	(195,584-226,562)	134,048	(131,119-138,578)
		**Dementia**	239,562	(236,955-242,468)	56,457	(53,952-58,606)
		**Frailty**	80,655	(80,230-81,183)	33,030	(32,789-33,302)
		**Dementia + frailty**	24,656	(24,405-24,990)	4,333	(4,131-4,502)
**75 and +**	**Male**	**N**	2,785,325		1,226,847	
		**MCI**	853,824	(829,085-888,544)	61,119	(59,320-63,151)
		**Dementia**	1,022,747	(1,011,254-1,035,423)	15,534	(14,660-16,325)
		**Frailty**	563,420	(562,878-564,862)	187,354	(186,841-187,841)
		**Dementia + frailty**	306,314	(303,344-307,943)	7,109	(6,605-7,622)
	**Female**	**N**	5,114,945		747,977	
		**MCI**	1,293,962	(1,242,553-1,338,705)	61,501	(59,625-63,079)
		**Dementia**	1,760,307	(1,749,279-1,768,741)	137,564	(136,656-138,727)
		**Frailty**	1,322,998	(1,321,352-1,324,930)	123,654	(123,125-123,962)
		**Dementia + frailty**	762,865	(759,795-765,688)	51,540	(51,140-51,872)

5th and 95th percentile ranges are provided in parentheses.

Projected number of dementia, mild cognitive impairment (MCI), and frailty by educational strata in 2043

		Year 2043	Less than high school	5th-95th percentile range	College and over	5th-95th percentile range
**60+ all**	**Male**	**N**	1,691,211	(1,690,191-1,692,839)	9,417,126	(9,415,255-9,419,752)
		**MCI**	348,837	(337,967-359,651)	217,452	(212,580-221,495)
		**Dementia**	509,333	(502,466-512,788)	73,290	(71,967-75,016)
		**Frailty**	281,074	(280,236-282,044)	697,211	(696,094-699,062)
		**Dementia + frailty**	182,741	(181,270-184,163)	32,907	(32,354-33,503)
	**Female**	**N**	1,890,567	(1,889,103-1,891,537)	11,229,800	(11,227,850-11,231,550)
		**MCI**	396,236	(387,027-407,217)	766,298	(758,495-781,365)
		**Dementia**	720,720	(717,453-723,694)	971,324	(965,840-978,319)
		**Frailty**	575,723	(574,930-577,389)	909,049	(907,720-910,726)
		**Dementia + frailty**	374,219	(371,977-375,550)	302,572	(301,213-303,704)
**60-74**	**Male**	**N**	832,377	(831,896-832,782)	5,815,982	(5,815,005-5,816,788)
		**MCI**	149,298	(141,314-154,761)	9,568	(8,420-10,591)
		**Dementia**	56,716	(54,774-60,335)	566	(403-783)
		**Frailty**	29,411	(29,119-29,635)	106,341	(105,818-106,983)
		**Dementia + frailty**	6,106	(5,900-6,297)	177	(121-266)
	**Female**	**N**	605,527	(605,324-605,891)	6,676,322	(6,675,451-6,676,829)
		**MCI**	47,226	(44,209-49,839)	337,871	(327,952-345,449)
		**Dementia**	69,458	(68,730-70,238)	139,243	(133,667-148,120)
		**Frailty**	21,144	(20,953-21,391)	90,890	(90,421-91,408)
		**Dementia + frailty**	5,768	(5,624-5,849)	8,677	(8,269-9,174)
**75 and +**	**Male**	**N**	858,821	(857,900-860,494)	3,601,326	(3,599,204-3,603,782)
		**MCI**	198,952	(194,157-207,704)	207,772	(203,320-212,481)
		**Dementia**	452,108	(446,588-455,389)	72,778	(71,488-74,339)
		**Frailty**	251,656	(251,032-252,556)	590,957	(589,867-592,621)
		**Dementia + frailty**	176,723	(175,370-177,883)	32,727	(32,197-33,289)
	**Female**	**N**	1,285,013	(1,283,571-1,286,028)	4,553,651	(4,551,988-4,555,035)
		**MCI**	349,841	(341,991-358,050)	429,248	(421,635-439,244)
		**Dementia**	651,498	(647,403-654,356)	831,635	(826,439-839,485)
		**Frailty**	554,595	(553,829-556,164)	818,096	(816,977-819,672)
		**Dementia + frailty**	368,456	(366,360-369,746)	293,985	(292,681-295,201)

5th and 95th percentile ranges are provided in parentheses.

Appendix Table 5. Total life expectancy and life expectancy with dementia, frailty, and both by education and sex strata

		Men				Women			
	Year	Total life expectancy	Life expectancy with dementia	Life expectancy with frailty	Life expectancy with dementia and frailty	Total life expectancy	Life expectancy with dementia	Life expectancy with frailty	Life expectancy with dementia and frailty
Less than high school	2016	18·42	4·06	2·21	1·08	23·53	5·58	3·86	2·17
	2025	19·50	4·84	2·50	1·31	24·36	5·49	4·12	2·11
	2034	19·53	4·89	2·56	1·32	24·30	5·66	4·23	1·98
	2043	19·68	5·04	2·64	1·39	24·12	5·93	4·38	2·08
High school	2016	18·67	1·21	1·67	0·35	23·77	4·04	3·70	1·73
	2025	19·43	1·52	1·83	0·47	24·75	3·85	4·11	1·82
	2034	19·51	1·53	1·83	0·46	24·75	3·63	4·09	1·64
	2043	19·56	1·50	1·86	0·45	24·73	3·66	4·17	1·64
College	2016	19·01	0·15	1·86	0·07	23·92	3·74	3·23	1·51
	2025	20·03	0·27	2·11	0·13	25·04	3·90	3·65	1·69
	2034	20·13	0·28	2·13	0·13	25·12	3·82	3·61	1·58
	2043	20·16	0·28	2·16	0·13	25·12	3·88	3·63	1·57

Appendix Figure 1. Expected life expectancy at age 65 years free of (A) dementia and (B) frailty, 2016–2043, by a static assumption model with reference to existing life expectancy estimation by the National Institute of Population and Social Security, Japan

A

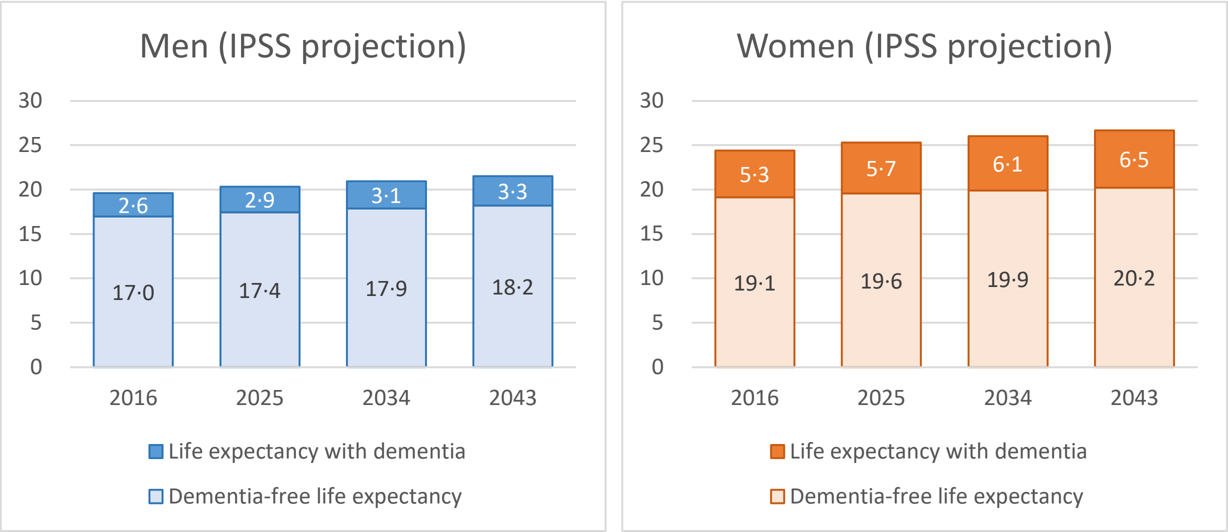

B

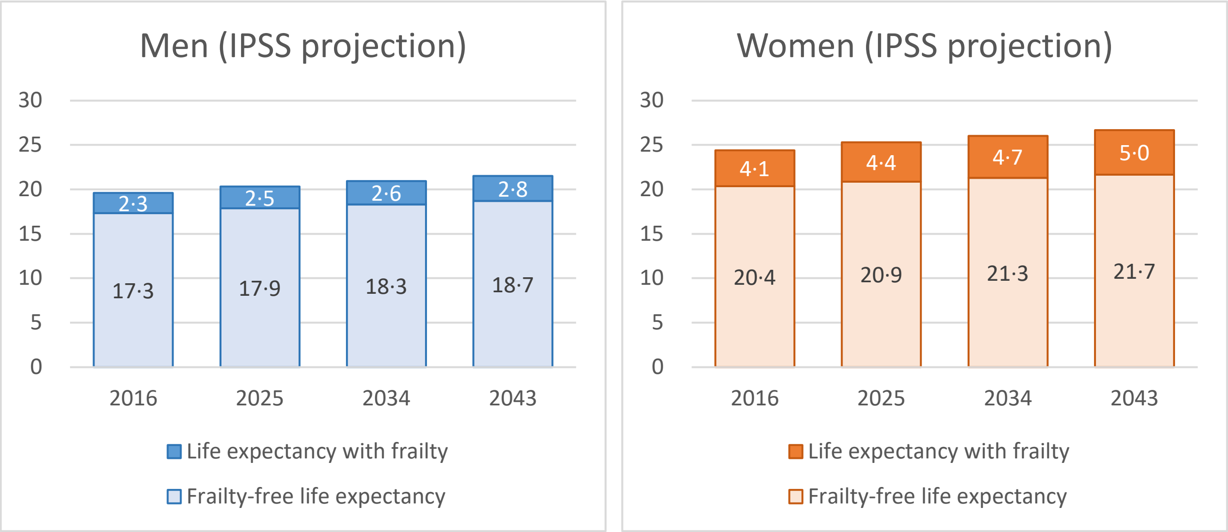

We assumed constant rates of age-sex-specific prevalence of dementia and frailty as of 2013. IPSS = National Institute of Population and Social Security Research. We downloaded population projection and lifetables from the URLs below:
http://www.ipss.go.jp/pp-zenkoku/j/zenkoku2017/db_zenkoku2017/s_tables/1-9.htm
http://www.ipss.go.jp/pp-zenkoku/j/zenkoku2017/db_zenkoku2017/s_tables/11-6.htm
The results indicate extension of life expectancy with dementia and frailty in both sexes, which leads to overestimation of future dementia and frailty prevalence compared with that produced by the current study.

Appendix Figure 2. Estimated education strata with reference to Barro-Lee data

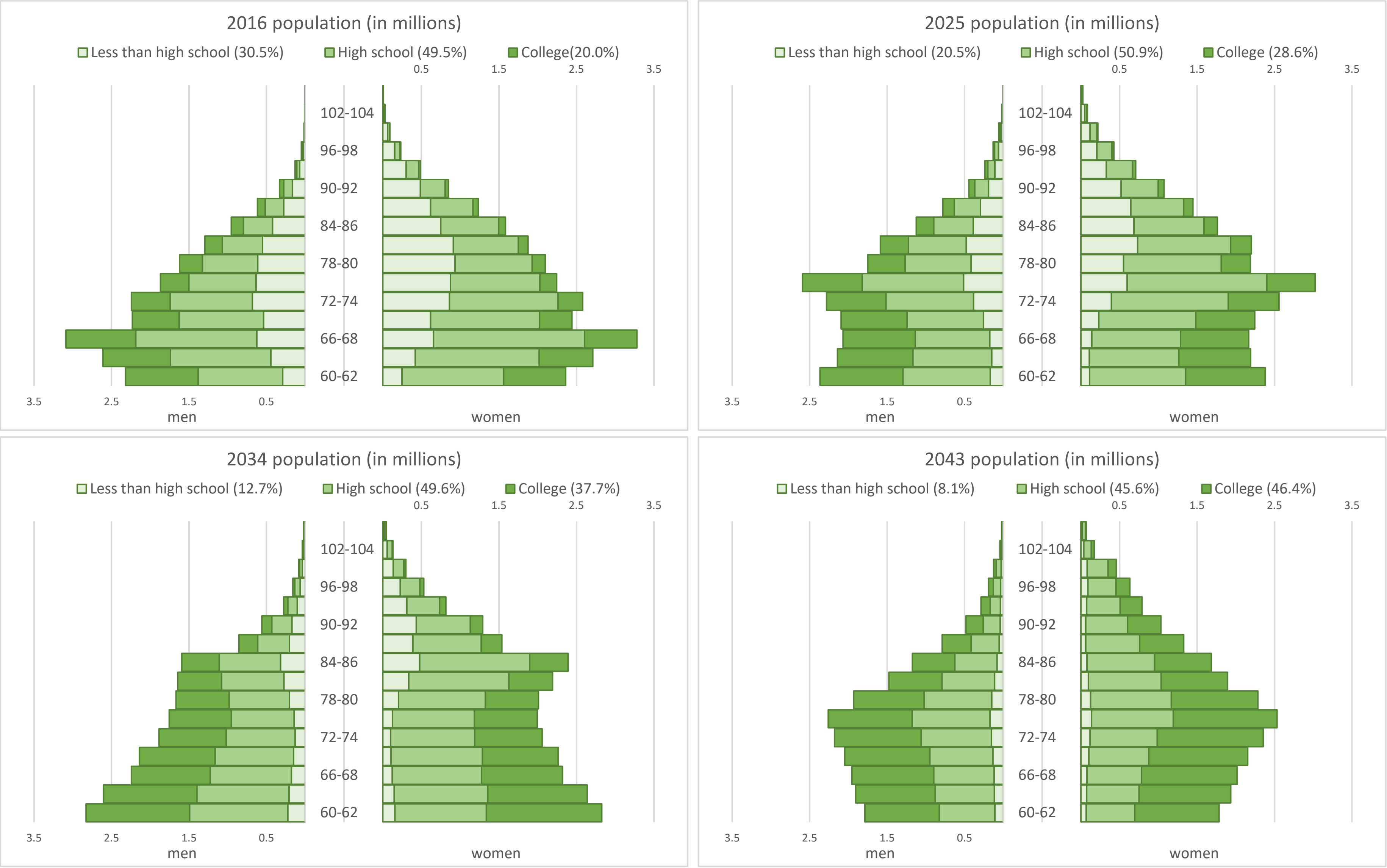

Appendix Table 6. Estimated education strata with reference to Barro-Lee data

Male	Primary (% of population)					Secondary (% of population)					Tertiary (% of population)				
Age group	Barro-Lee 2010	JFEM 2016	JFEM 2025	JFEM 2034	JFEM 2043	Barro-Lee 2010	JFEM 2016	JFEM 2025	JFEM 2034	JFEM 2043	Barro-Lee 2010	JFEM 2016	JFEM 2025	JFEM 2034	JFEM 2043
30–34 years	6·18					44·47					49·28				
35–39 years	7·83					47·81					44·30				
40–44 years	7·77					47·84					44·33				
45–49 years	8·07					49·58					42·26				
50–54 years	8·09					49·55					42·27				
55–59 years	16·73					56·46					26·72				
60–64 years	16·74	14·11	7·06	7·94	6·40	56·37	48·07	47·58	45·14	40·01	26·80	37·82	45·36	46·92	53·59
65–69 years	25·56	20·39	8·70	7·83	7·24	58·97	50·23	46·52	46·57	41·89	15·37	29·39	44·79	45·60	50·87
70–74 years	24·27	28·58	15·45	6·92	7·86	59·73	47·67	48·70	47·30	45·19	15·90	23·75	35·85	45·77	46·94
75+ years	29·82	40·41	28·68	17·97	10·29	58·38	41·79	46·96	47·33	46·53	11·65	17·80	24·36	34·71	43·18
**Female**	**Primary (% of population)**					**Secondary (% of population)**					**Tertiary (% of population)**				
**Age group**	**Barro-Lee 2010**	**JFEM 2016**	**JFEM 2025**	**JFEM 2034**	**JFEM 2043**	**Barro-Lee 2010**	**JFEM 2016**	**JFEM 2025**	**JFEM 2034**	**JFEM 2043**	**Barro-Lee 2010**	**JFEM 2016**	**JFEM 2025**	**JFEM 2034**	**JFEM 2043**
30–34 years	2·10					38·66					59·18				
35–39 years	3·87					46·83					49·23				
40–44 years	5·00					46·28					48·65				
45–49 years	5·49					53·86					40·56				
50–54 years	5·49					53·86					40·56				
55–59 years	15·77					64·28					19·85				
60–64 years	15·77	12·24	4·86	5·63	4·31	64·28	56·94	52·28	43·02	35·35	19·85	30·82	42·86	51·35	60·34
65–69 years	27·57	20·10	6·70	5·23	5·16	64·73	59·28	53·11	49·52	37·36	7·57	20·62	40·18	45·25	57·48
70–74 years	26·36	31·08	13·82	4·89	5·54	65·81	55·24	58·05	52·52	44·46	7·70	13·68	28·13	42·59	50·00
75+ years old	33·79	47·67	33·78	20·43	10·19	62·32	45·36	53·11	55·64	53·67	3·57	6·97	13·11	23·93	36·14

Note. We compared the education distributions of people 60–64 years old in 2025, 2034, and 2043 with those 45–49 years old, 35–44 years old, and 30–34 years old in Barro-Lee 2010 data. Barro RJ, Lee JW. A new data set of educational attainment in the world, 1950–2010. *Journal of development economics*. 2013;**104**:184–98.

Appendix Figure 3. Population pyramid by cognitive functional status (A) and frailty status (B) corresponding to Table 1.

A

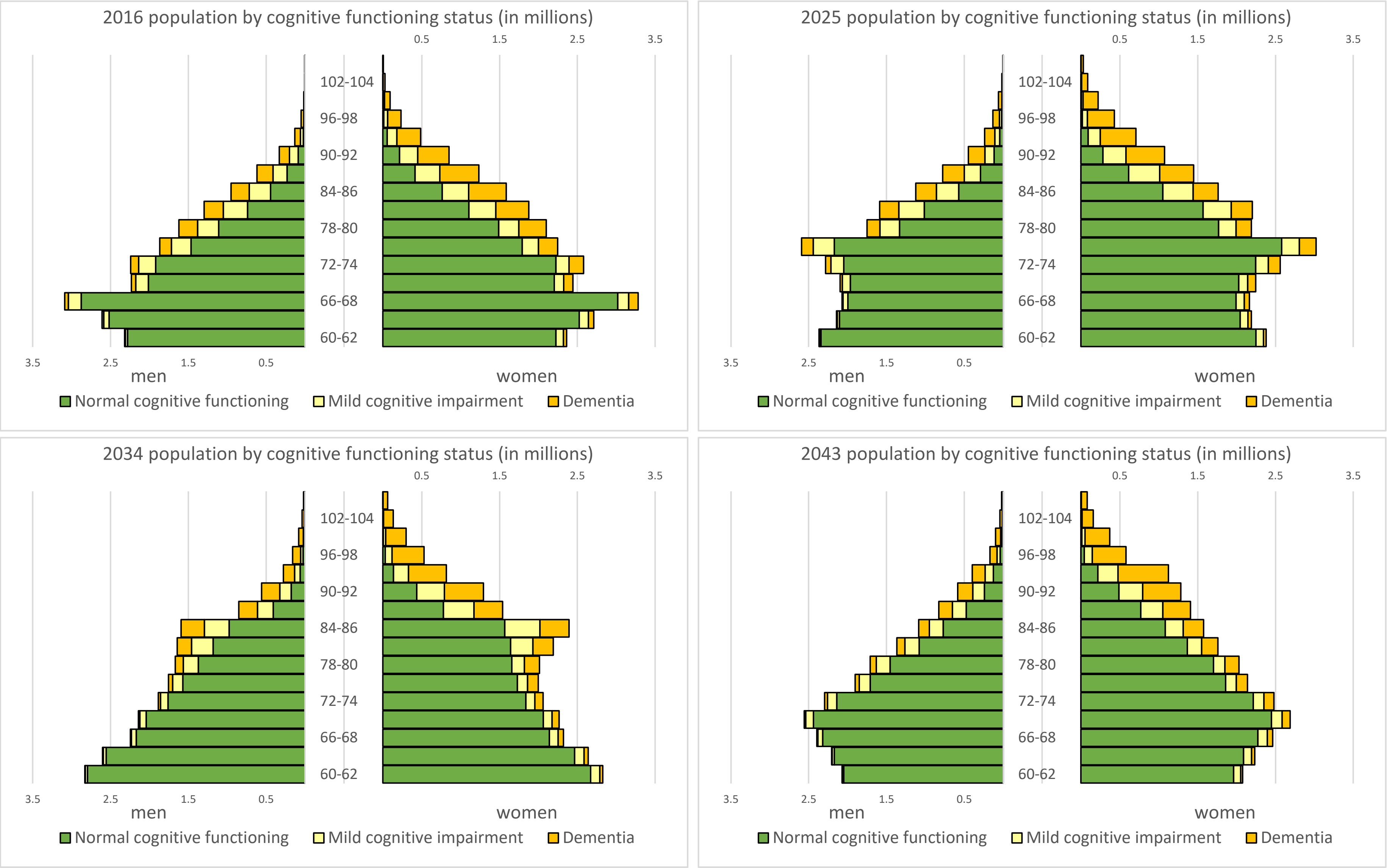

B

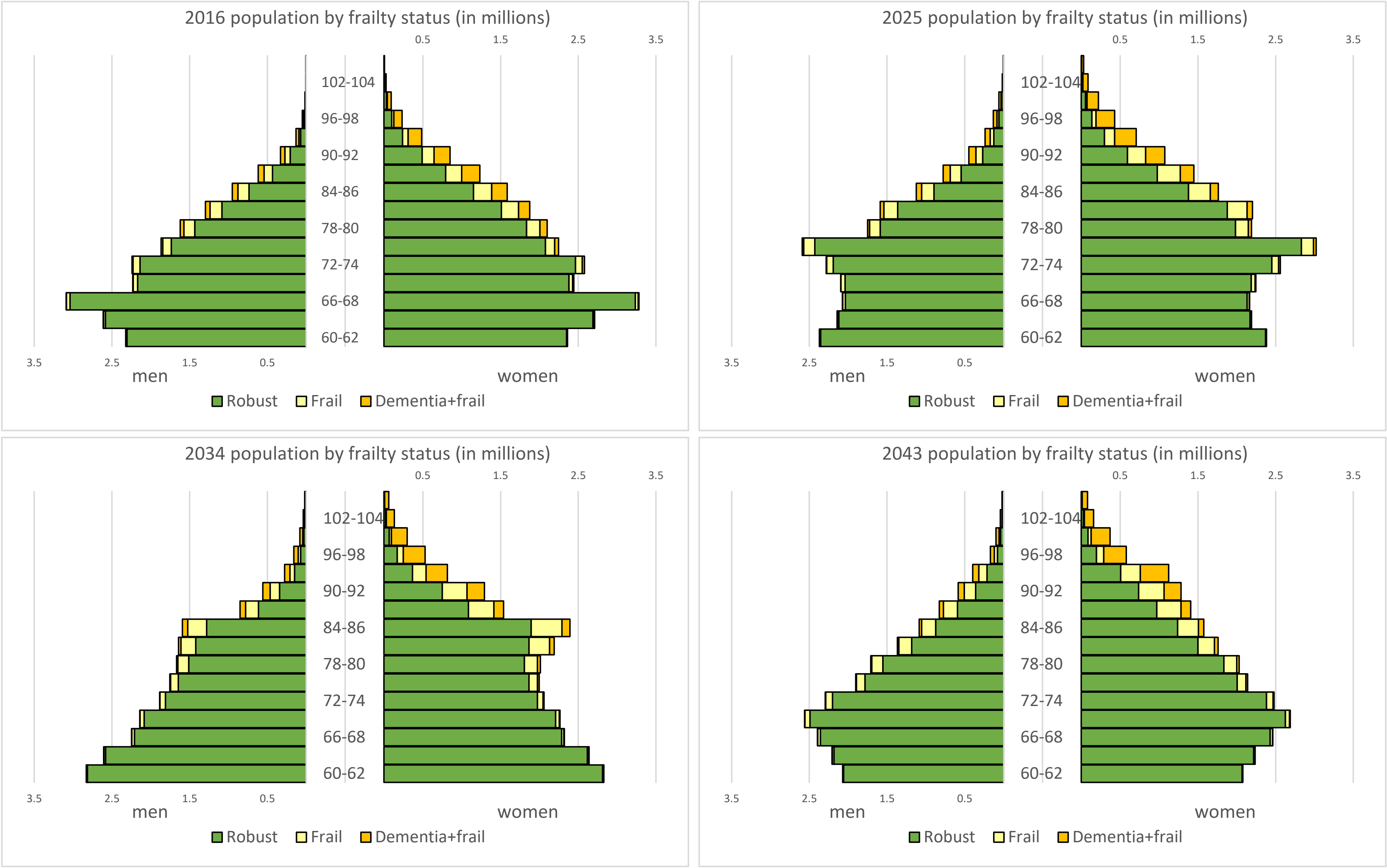
2016 population by frailty status (in millions)
2025 population by frailty status (in millions)
2034 population by frailty status (in millions)
2043 population by frailty status (in millions)
0.5
1.5
2.5
3.5
102-104
96-98
90-92
84-86
78-80
72-74
66-68
60-62
men
women
Robust
Frail
Dementia+frail
